# Giant Hydrogen Sulfide Plume in the Oxygen Minimum Zone off Peru Supports Chemolithoautotrophy

**DOI:** 10.1371/journal.pone.0068661

**Published:** 2013-08-21

**Authors:** Harald Schunck, Gaute Lavik, Dhwani K. Desai, Tobias Großkopf, Tim Kalvelage, Carolin R. Löscher, Aurélien Paulmier, Sergio Contreras, Herbert Siegel, Moritz Holtappels, Philip Rosenstiel, Markus B. Schilhabel, Michelle Graco, Ruth A. Schmitz, Marcel M. M. Kuypers, Julie LaRoche

**Affiliations:** 1 Research Division Marine Biogeochemistry, GEOMAR Helmholtz Centre for Ocean Research Kiel, Kiel, Germany; 2 Institute for General Microbiology, Christian-Albrechts-University, Kiel, Germany; 3 Department of Biogeochemistry, Max-Planck-Institute for Marine Microbiology, Bremen, Germany; 4 Department of Biology, Dalhousie University, Halifax, Nova Scotia, Canada; 5 College of Engineering, Mathematics and Physical Sciences, University of Exeter, Exeter, United Kingdom; 6 Laboratory for Studies in Geophysics and Spatial Oceanography, Institute of Research for Development, Toulouse, France; 7 Dirección de Investigaciones Oceanográficas, Instituto del Mar del Perú, Callao, Peru; 8 Large Lakes Observatory, University of Minnesota Duluth, Duluth, Minnesota, United States of America; 9 Physical Oceanography and Instrumentation, Leibniz Institute for Baltic Sea Research Warnemünde, Rostock, Germany; 10 Institute of Clinical Molecular Biology, Christian-Albrechts-University, Kiel, Germany; Royal Netherlands Institute of Sea Research (NIOZ), The Netherlands

## Abstract

In Eastern Boundary Upwelling Systems nutrient-rich waters are transported to the ocean surface, fuelling high photoautotrophic primary production. Subsequent heterotrophic decomposition of the produced biomass increases the oxygen-depletion at intermediate water depths, which can result in the formation of oxygen minimum zones (OMZ). OMZs can sporadically accumulate hydrogen sulfide (H_2_S), which is toxic to most multicellular organisms and has been implicated in massive fish kills. During a cruise to the OMZ off Peru in January 2009 we found a sulfidic plume in continental shelf waters, covering an area >5500 km^2^, which contained ∼2.2×10^4^ tons of H_2_S. This was the first time that H_2_S was measured in the Peruvian OMZ and with ∼440 km^3^ the largest plume ever reported for oceanic waters. We assessed the phylogenetic and functional diversity of the inhabiting microbial community by high-throughput sequencing of DNA and RNA, while its metabolic activity was determined with rate measurements of carbon fixation and nitrogen transformation processes. The waters were dominated by several distinct γ-, δ- and ε-proteobacterial taxa associated with either sulfur oxidation or sulfate reduction. Our results suggest that these chemolithoautotrophic bacteria utilized several oxidants (oxygen, nitrate, nitrite, nitric oxide and nitrous oxide) to detoxify the sulfidic waters well below the oxic surface. The chemolithoautotrophic activity at our sampling site led to high rates of dark carbon fixation. Assuming that these chemolithoautotrophic rates were maintained throughout the sulfidic waters, they could be representing as much as ∼30% of the photoautotrophic carbon fixation.

Postulated changes such as eutrophication and global warming, which lead to an expansion and intensification of OMZs, might also increase the frequency of sulfidic waters. We suggest that the chemolithoautotrophically fixed carbon may be involved in a negative feedback loop that could fuel further sulfate reduction and potentially stabilize the sulfidic OMZ waters.

## Introduction

Eastern Boundary Upwelling Systems are found along the westward shelves of the continents in both the Atlantic and the Pacific Ocean. They are characterized by high photoautotrophic primary production, which is driven by the upwelling of nutrient-rich waters [1]. The produced biomass supports large fish populations in these regions, underlining the importance of Eastern Boundary Upwelling Systems in providing a source of food for mankind [2]–[6]. However, a significant proportion of the produced biomass also sinks through the water column and is remineralized in subsurface waters, contributing to the oxygen (O_2_) depletion in intermediate water depths of these regions. Combined with large-scale ocean circulation patterns and poor ventilation of intermediate waters with the ocean surface, the remineralization of rich organic matter leads to oxygen-depleted zones of varying intensities [7]–[10]. These oxygen-depleted waters, also referred to as oxygen minimum zones (OMZs), are found in the eastern tropical North and South Pacific, and to a lesser extent in the eastern tropical North and South Atlantic [11]. In addition to OMZs found in regions of strong upwelling, oxygen-depleted waters are also present in the northern Indian Ocean and in enclosed basins like the Baltic and the Black Sea [12].

The OMZ off Peru, Chile and Ecuador in the South Pacific Ocean is the largest oceanic area where O_2_ concentrations are reported to fall below the detection limit of the most sensitive O_2_ sensors (∼10–100 nM) [13]–[16]. In the absence of O_2_, organic carbon degradation has been historically attributed to heterotrophic denitrification, the reduction of nitrate (NO_3_^−^) to dinitrogen gas (N_2_) [17]–[19]. Some *in situ* experiments have shown active heterotrophic denitrification in OMZ waters [20], [21], however numerous studies have demonstrated that anammox, the anaerobic oxidation of ammonium (NH_4_^+^) with nitrite (NO_2_^−^) to N_2_, is responsible for the major loss of fixed nitrogen from the OMZ off Namibia [22], Oman [15], Peru [23]–[25] and Chile [26]. As anammox is an autotrophic process, its dominance over heterotrophic denitrification questions our understanding of organic matter remineralization in OMZ waters. This is supported by the hypothesis that oxygen-depletion leads to a shift of the microbial community from organoheterotrophs to chemolithotrophs [27], [28].

In anoxic sediments, the main process for the degradation of organic carbon is considered to be microbial sulfate (SO_4_^2−^) reduction to elemental sulfur (S^0^) and hydrogen sulfide (H_2_S) [29]. The release of large quantities of the toxic H_2_S from these underlying sediments can lead to the occasional build-up of high concentrations of H_2_S in bottom waters [30]–[33]. On the other hand, it has also been suggested that H_2_S build-up in oceanic waters could be caused by SO_4_^2−^ reduction of pelagic microorganisms within the water column [34], [35]. A recent study suggested that an active, but cryptic sulfur cycle is present in non-sulfidic subsurface waters in the eastern tropical South Pacific OMZ off northern Chile [14]. According to this hypothesis, SO_4_^2−^ reduction and consequently H_2_S formation would take place in the water column, even when thermodynamically more favourable electron acceptors like NO_3_^−^ or NO_2_^−^ are still present. However, the resulting H_2_S would be rapidly re-oxidized to S^0^ or SO_4_^2−^, such that the two processes are in steady-state, thereby preventing an accumulation of H_2_S [31], [36].

The presence of chemolithoautotrophic γ-proteobacteria involved in sulfur cycling (e.g. related to the uncultured SUP05 cluster bacterium, referred to henceforth as SUP05) in non-sulfidic OMZ waters supports the hypothesis of a cryptic sulfur cycle [8], [14], [37]–[40]. Studies conducted during occurrences of sulfidic events in the Benguela Current upwelling OMZ and in a seasonally anoxic fjord in Canada demonstrated that γ-proteobacteria related to SUP05 are highly abundant in sulfidic waters and that they probably detoxified the waters via the chemolithotrophic oxidation of H_2_S coupled to the reduction of NO_3_^−^ [31], [41], a pathway termed sulfur-driven autotrophic denitrification. In addition, chemolithoautotrophic α- and especially ε-proteobacteria detected in the sulfidic waters in the Benguela Current upwelling OMZ as well as in the sulfidic zones of the Baltic and the Black Sea could also be important members of microbial communities in sulfidic plumes [31], [42], [43].

The initiation, termination and frequency of sulfidic events in oceanic OMZs are so far poorly understood, and H_2_S in the water column has been mostly observed in enclosed basins like the Baltic Sea [43]–[45], the Black Sea [46]–[48], the Cariaco trench off Venezuela [49], [50] and the Saanich Inlet in Canada [41], [51]. With the exception of a few studies mentioning the characteristic odor of H_2_S and anecdotal reports of Peruvian fishermen on ‘black’ fishing gear in relation to the so-called ‘aquajes’ conditions for the OMZ off Peru, sulfidic waters have not been measured in the Pacific Ocean so far [31], [34], [35], [52].

Negative consequences on fish stocks and quality of life along the populated coastal upwelling regions are potentially severe, because H_2_S is highly toxic to animals and humans and has already been invoked as the cause for occasional but massive fish kills in African shelf waters [53]–[55]. The anticipated decrease in O_2_ concentrations and the increase in water column stratification, as predicted from global change [11], as well as local eutrophication [27], [35], might lead to more frequent and intense depletion of O_2_ and of alternate electron acceptors (e.g. NO_3_^−^ and NO_2_^−^), favouring the development of sulfidic waters within OMZs [31]. Given that the detoxification of sulfidic water is a microbial process, it is important to assess the phylogenetic structure and the metabolic response of the endemic microbial community to the accumulation of H_2_S.

We explored the microbial community structure and its transcriptional activity in such a sulfidic event off the coast of Peru with high-throughput metagenomic and metatranscriptomic sequencing. We present rate measurements of carbon dioxide (CO_2_) fixation and nitrogen transformation processes as well as total bacterial cell counts with flow-cytometry. Several of the proteobacterial taxa that were dominant in the sulfidic waters were expressing genes involved in the sulfur cycle, which reflected various metabolic strategies for H_2_S oxidation. Our data further suggests that the microbial communities were responsible for considerable light-independent CO_2_ fixation.

## Results and Discussion

### Description of the sampling site

**Figure 1:**
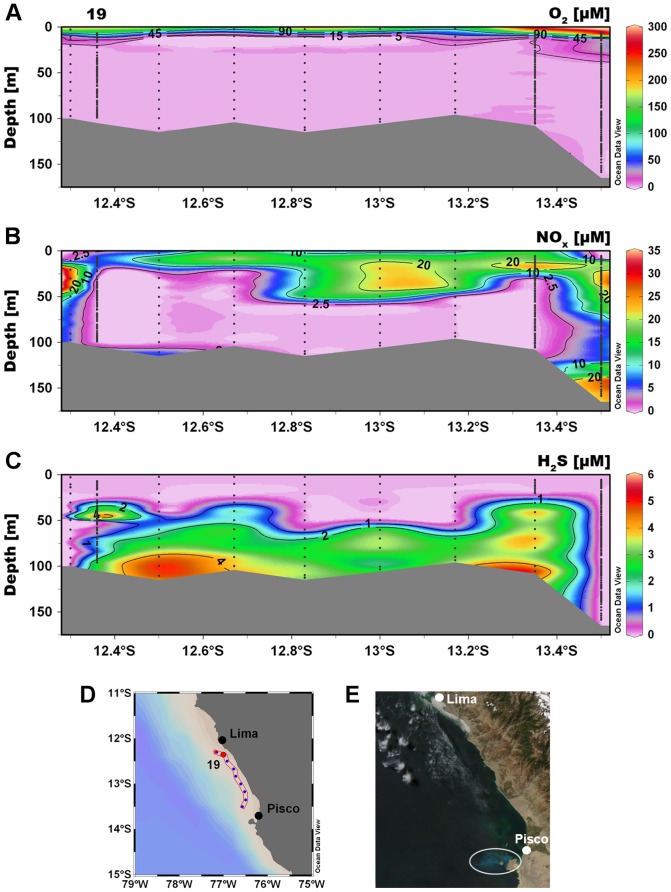
Extent of the sulfidic plume off the Peruvian coast. (A) Vertical distribution of O_2_ concentrations. (B) Vertical distribution of NO_x_ (the sum of NO_3_^−^ and NO_2_^−^) concentrations. (C) Vertical distribution of H_2_S concentrations. (D) Areal view of stations sampled along the transect off the Peruvian coast between Lima and Pisco. The station (19) that was analyzed in detail is marked with a red dot. (E) Satellite image (MODIS) showing a colloidal S^0^ plume (white circle) on May 8^th^, 2009.

During RV Meteor cruise M77/3 on the Peruvian shelf in January 2009 we found sulfidic waters stretching from Lima to Paracas National Reserve southwest of Pisco (Figure 1). O_2_ concentration in shelf waters in the study area were generally below the detection limit of our microsensor (0.5–1 µM) at water depth below 20 m (from 12° S to 14° S; Figure 1A), while NO_x_ (the sum of NO_3_^−^ and NO_2_^−^) was heavily depleted in the water column throughout the transect from 20–60 m to the bottom (from 12°30′S to 13°50′S; Figure 1B), mirror-imaging the distribution of H_2_S (Figure 1C). Sulfidic waters were first detected on January 9^th^ south of Lima and seemed to have persisted until the end of the cruise, when H_2_S-containing waters covered ∼5500 km^2^ of the shelf. The thickness of the sulfidic layer was about 80 m (Figure 1C), yielding the largest sulfidic plume (∼440 km^3^) ever reported for oceanic waters. We calculated a H_2_S content of ∼2.2×10^4^ tons. The total area affected by H_2_S may have been even larger, as we did not map the extent of the sulfidic plume into the protected area of the Paracas National Reserve.

Remote satellite sensing revealed large patches (50–150 km^2^) of turquoise discoloured surface-waters, attributable to the formation of colloidal S^0^ upon H_2_S oxidation [56], [57] off Lima as well as in Paracas National Reserve during our sampling campaign (Figure S1). The larger extension of H_2_S in deeper waters when compared to the colloidal S^0^ in the surface indicated that most of the H_2_S was oxidized in subsurface waters, similar to the observations from the Benguela upwelling system [31]. Colloidal S^0^ plumes measuring up to 500 km^2^ were observed in the same region again in May 2009, indicating that the occurrence of S^0^ plumes detected in January 2009 was not a unique event (Figure 1E). This suggests that sulfidic waters in the OMZ off Peru might be more frequent and persistent than originally thought.

**Figure 2:**
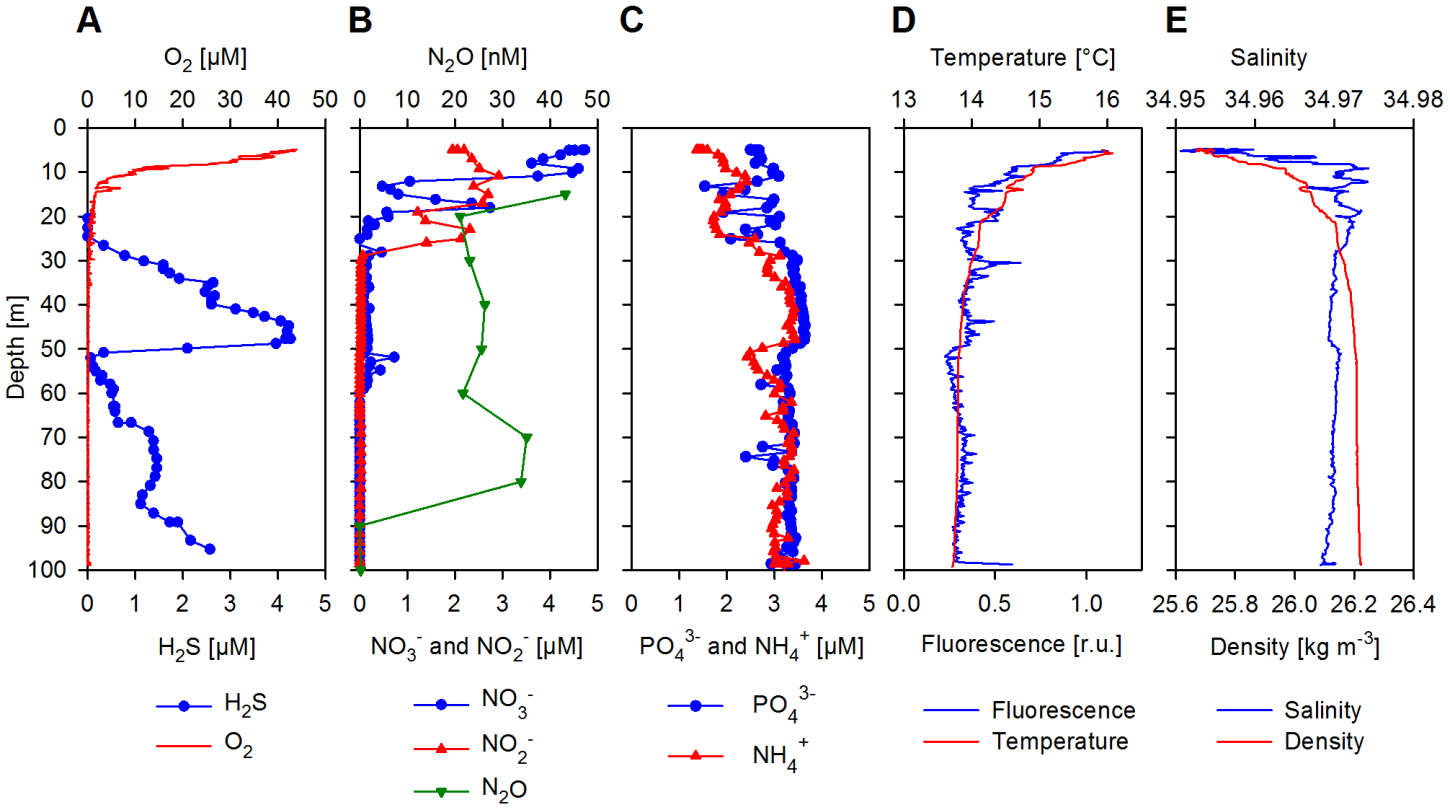
Vertical distribution of physical, chemical and biological water properties. (A) Concentrations of H_2_S and O_2_. (B) Concentrations of NO_3_^−^, NO_2_^−^ and N_2_O. (C) Concentrations of PO_4_^3−^ and NH_4_^+^. (D) *In situ* fluorescence (chlorophyll, relative units as measured with the pump-CTD) and temperature. (E) Salinity and density.

A vertical profile of the sulfidic water column (station 19), sampled during the upcast with a pump-CTD on January 9^th^, 2009 at a site located ∼15 km offshore Lima (12°21.88′S, 77°0.00′W, ∼100 m water depth; Figure 1D) was the target of a detailed analysis. The surface mixed layer was shallow with the thermocline at about 10 m water depth (Figure 2D). The surface temperature (>16°C) was only ∼2°C warmer than the bottom waters and the salinity (34.95–34.97) changed merely slightly with depths, which indicated an active upwelling of subsurface waters. Even the surface waters were characterized by low O_2_ conditions down to 40 µM (or ∼15% saturation; Figure 2A). During the downcast, O_2_ decreased at the thermocline and dropped to about the detection limit of our amperometric O_2_ microsensor (0.5–1 µM) at around 20 m. Nevertheless, trace amounts of O_2_ (<1 µM) were still detected with some variability down to ∼40 m water depth. These low concentrations of O_2_ were close to detection limit of our sensor and we cannot rule out whether this was due to water advection caused by the CTD rosette or to a memory effect of the sensor. However, using a highly-sensitive self-calibrating Switchable Trace amount Oxygen (STOX) sensor with a detection limit of ∼50 nM [13], [16] during the upcast of the CTD rosette, O_2_ was undetectable below 20 m, and therefore we defined this zone as anoxic. We detected large vertical movement in the oxycline from 5–18 m due to internal waves, which may have caused non steady state conditions and induced a flux of O_2_ down into the anoxic waters.

Phosphate (PO_4_^3−^) and NH_4_^+^ concentrations remained high (both around 3 µM) and stable throughout the water column, with NH_4_^+^ only having a minor drop in concentrations near 50 m (Figure 2C). NO_3_^−^ concentrations (detection limit ∼0.1 µM) were lower than those measured in the southern part of the study area and lower than expected from strong upwelling regions in general. Highest NO_3_^−^ concentrations with ∼5 µM were found in surface waters, but dropped rapidly below1 µM, just beneath the oxycline at ∼19 m (Figure 2B). Detectable concentrations of NO_3_^−^ (ranging from 0.1–0.2 µM) were measured down to 59 m with the exception of 52–55 m, where a small increase up to 0.7 µM was observed. NO_2_^−^ (detection limit ∼0.01 µM) was high (1.5–3 µM) in surface waters down to 26 m. Trace concentrations (∼25 nM) were measurable down to 50 m and again from 67–81 m. Nitrous oxide (N_2_O) concentrations ranged between 20–40 nM from 15–80 m and dropped below the detection limit closer to the bottom of the water column (Figure 2B). H_2_S was first detected (with both microsensor and wet chemistry) at 26 m and increased steadily, reaching a concentration of 4.2 µM at 48 m (Figure 2A). This maximum was followed by a rapid drop in concentrations to below 0.1 µM at 52–53 m, before increasing again to about 2.6 µM at 95 m, approximately 5 m above the sediment. H_2_S concentrations directly at the sediment-water interface were probably even greater, but were not measured in this study.

### Phylogenetic diversity of the microbial community

Based on the monitoring of O_2_ and H_2_S during the upcast, we defined three zones within the water column, where we carried out a detailed sampling: the oxic surface (5 m sample, where sampling was stopped when internal waves decreased O_2_ concentrations to below 30 µM), the upper boundary of the anoxic zone (15 and 20 m samples) and the sulfidic zone (30, 40, 50, 60, 80 and 100 m samples). Both a hierarchical clustering approach with a statistical analysis of taxonomic assignments and a non-parametric Multidimensional Scaling indicated that the selected sample groups were justified (an ANOSIM test using a Bray-Curtis distance measure showed a Global R value of 0.83 and a significance level of 0.1%, Figure S2).

**Figure 3:**
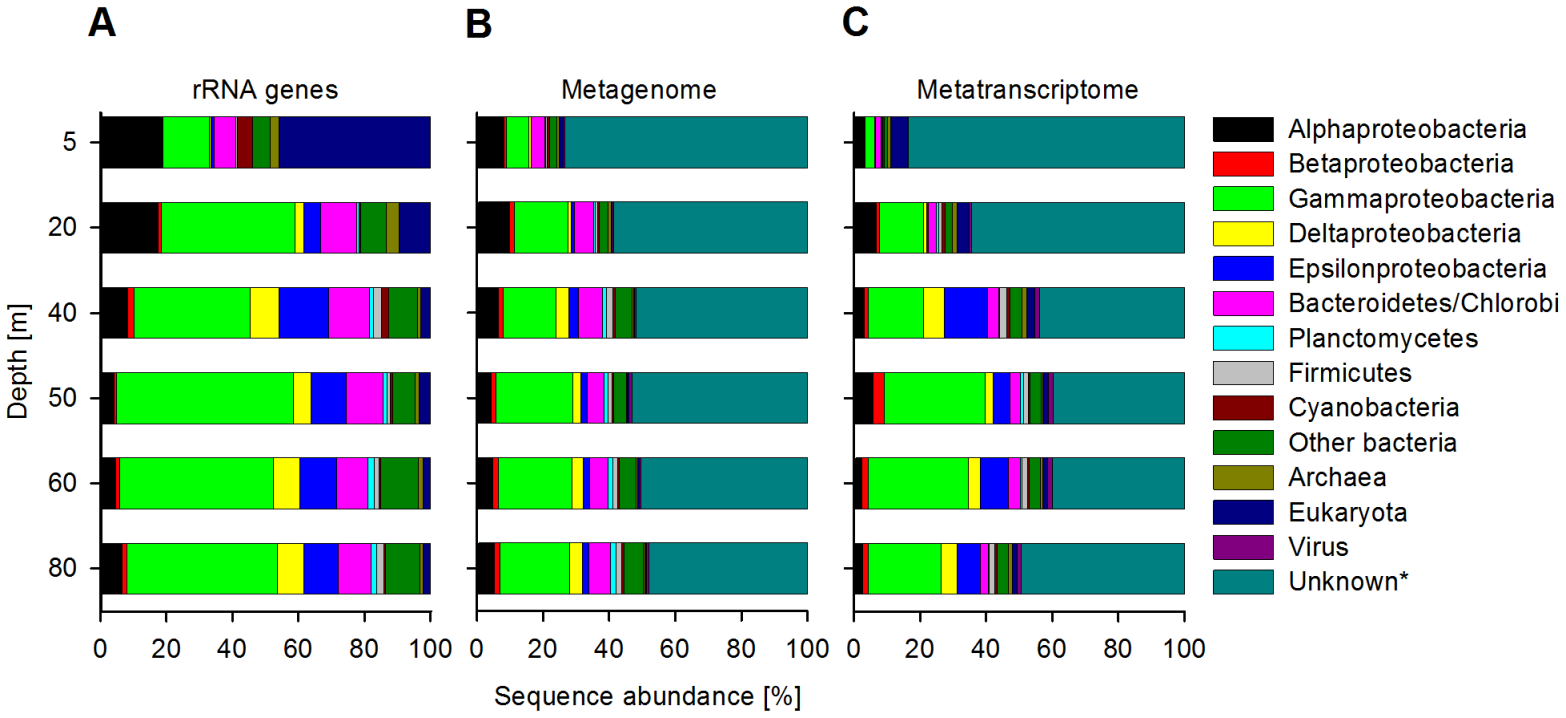
Vertical distribution of taxonomic assignments. Shown on either domain, phylum or class level. (A) rRNA genes in percent of all rRNA genes (5,923 sequences). (B) Metagenomic sequences in percent of all metagenomic sequences (1,882,842 sequences, excluding rRNA genes). (C) Metatranscriptomic sequences in percent of all metatranscriptomic sequences (421,528 sequences, excluding rRNAs). ‘Other Bacteria’ include Acidobacteria, Actinobacteria, Aquificae, Chlamydiae, Chloroflexi, Deferribacteres, Deinococcus-Thermus, Dictyoglomi, Elusimicrobia, Fibrobacteres, Fusobacteria, Gemmatimonadetes, Lentisphaerae, Nitrospirae, Spirochaetes, Synergistetes, Tenericutes, Thermotogae and Verrucomicrobia. *Sequences with no significant match against the non-redundant database of NCBI or sequences with a match that lacks taxonomic information.

Using a 98% similarity cut off, the metagenomes and metatranscriptomes accounted for an average of 263,606 (DNA) and 98,785 (RNA) unique sequences (clusters) per depths (Table S1). A total of 4809 (DNA) and 3872 (RNA) different taxa were identified using BLAST-searches, revealing a highly diverse microbial community at all depths (Table S2). The taxonomic composition based on all rRNA genes (5,923 sequences) using BLASTn-searches against the SILVA database is shown in Figure 3A and based on the metagenomes (1,882,842 sequences, excluding all rRNA genes) and the metatranscriptome (421,528 sequences, excluding all rRNAs) using BLASTx-searches against the non-redundant database of NCBI in Figure 3B and 3C. A large percentage of the sequences found in both the metagenomes and metatranscriptomes had no significant match against the non-redundant database of NCBI. On average, 49% of the sequences remained unidentified, which is comparable to other studies that utilized high-throughput sequencing technologies in marine habitats [37], [40], [58]–[60].

The community structure presented a stable and uniform distribution at the phylum-level, especially within the sulfidic zone (Figure 3). The metagenome data suggested that the microbial community was overall dominated by proteobacteria (16.6–34.1% of all DNA sequences, including all unidentified sequences); while the Bacteroidetes/Chlorobi-group was the second largest group we could identify (3.9–7.4%). In oxic and anoxic waters, both α- and γ-proteobacterial sequences were abundant (6.9–16.4%), similar to previous findings from the OMZs off northern Chile [38] and the Arabian Sea [61]. In sulfidic waters, γ-proteobacteria were clearly dominating (up to 23.2%) and we further found a significant increase in the frequencies of δ- and ε-proteobacterial sequences (1.9–4.0%), which were much less abundant in the 5 and 20 m samples. The δ- and ε-proteobacterial sequences were even more abundant within the rRNA gene dataset (Figure 3A) when compared to the metagenomes (Figure 3B), probably due to the lack of representatives genomes for these groups.

The metatranscriptomes showed a more variable picture of the microbial community (Figure 3C). In surface waters eukaryotic sequences formed the largest identifiable group (5%), while at all other depths γ-proteobacteria were dominating (13.3–30.5%). In sulfidic waters ε-proteobacterial transcripts were further identified in relatively high, but also more variable proportions (5.0–13.2%), when compared to the metagenomes. Notably, ‘other bacteria’, summarizing 19 bacterial phyla, were present at all depth, but never exceeded 5.8% of the sequences, in both the metagenome and the metatranscriptome datasets.

**Figure 4:**
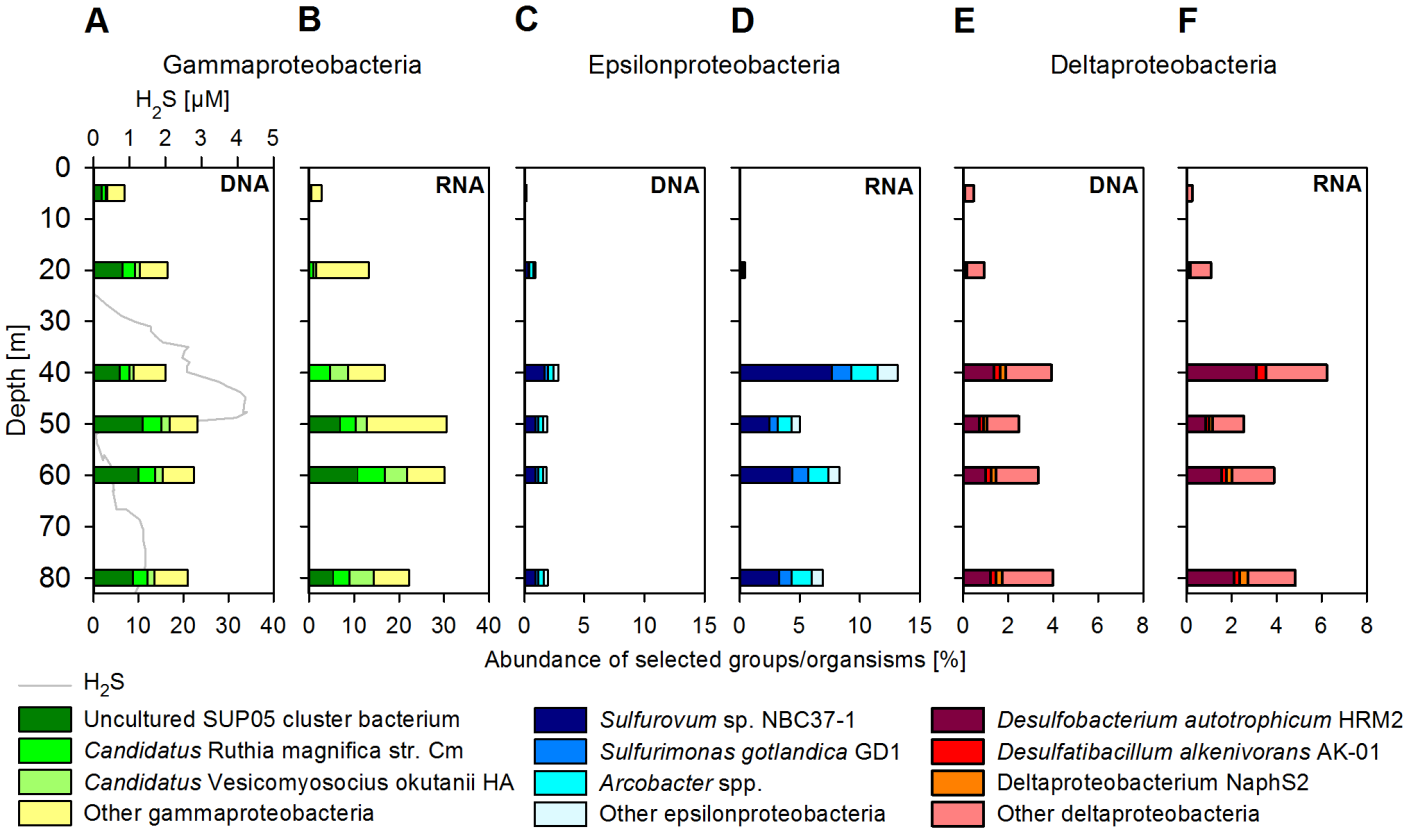
Vertical distribution of dominant proteobacterial taxa. Shown in percent of all sequences (excluding rRNA genes and rRNAs). (A) DNA and (B) RNA sequence abundances for γ-proteobacteria. (C) DNA and (D) RNA sequence abundances for ε-proteobacteria. (E) DNA and (F) RNA sequence abundances for δ-proteobacteria.

A more detailed analysis of the metagenomes revealed that the oxic surface waters harboured several different photoheterotrophic organisms (Figure S3). Prokaryotes similar to *Candidatus* Pelagibacter sp. HTCC7211 accounted for 1.6% and relatives of *Candidatus* Pelagibacter sp. HTCC1002 made up 0.4% of all DNA sequences, which is in agreement with other studies conducted in OMZs [14], [37], [40], [61]. Additionally, photosynthetic *Synechococcus* spp., which are also known to be present in OMZ waters [62] accounted for about 0.4% of the sequences. However, the most abundant single taxon identified in the oxic surface metagenome had high similarity to the uncultured SUP05 cluster bacterium (1.9%), a chemolithoautotrophic sulfur oxidizer, which has been detected previously in oxygen-depleted [14], [37], [38], [40] and sulfidic waters [31], [41], [42], [63]. In the metagenome sample from the anoxic zone (20 m), SUP05 was also the dominant taxon with 6.6% of all DNA sequences (Figure 4A and S3). At 20 m and below, γ-proteobacterial sulfur oxidizers (GSO) related to gill symbionts of deep-sea hydrothermal-vent clams, *Candidatus* Ruthia magnifica str. Cm (2.6%) and *Candidatus* Vesicomyosocius okutanii HA (1.4%) [64], [65] became increasingly abundant. This indicated that the GSO-community at our sampling site was composed of at least three separate taxa. In the sulfidic zone, the dominance of the GSO-group was even higher, reaching a maximum of 17% of all DNA sequences at 50 m (Figure 4 and S3). Other common microorganisms in the metagenome were similar to the ε-proteobacterium *Sulfurovum* sp. NBC37- 1 (up to 1.7%) and to the δ-proteobacterium *Desulfobacterium autotrophicum* HRM2 (up to 1.4%) [66]–[68]. In all sulfidic depths, organisms related to SUP05, *R. magnifica*, *V. okutanii*, *Sulfurovum* and *D. autotrophicum* were the five most abundant organisms that we identified with BLAST-searches.

In contrast to the metagenomes, the metatranscriptomes reflect the suite of genes that were expressed in the microbial community at the time of sampling and therefore displayed a more variable picture of the microbial community, showing to some extent its actual metabolic activity. In the oxic surface waters, we found RNA sequences similar to the archaeal ammonia-oxidizer *Nitrosopumilus maritimus* SCM1 and the α-proteobacterium *Magnetospirillum gryphiswaldense* to comprise the largest single taxa (0.7 and 0.4%). *N. maritimus* is considered a ‘classical’ inhabitant of the oxycline in OMZ waters [14], [37], [39], [40]. At 20 m, RNA sequences similar to the γ-proteobacteria *Marinomonas* sp. MWYL1 and *Neptuniibacter* c*aesariensis* were most abundant (1.3 and 1.2%).

Similar to the metagenome assignments, the GSO-group was predominant in the metatranscriptomes throughout the sulfidic zone. However, the composition of the GSO-group was variable and changed with depths. While SUP05 was virtually undetectable using BLAST-searches in the 5, 20 and 40 m metatranscriptomes (0–0.1%; Figure 4B and S3), GSO-related RNA sequences at these depths could be almost exclusively assigned to the relatives of *R. magnifica* and *V. okutanii*. In all other metatranscriptomes from sulfidic waters (50, 60 and 80 m), sequences similar to SUP05 represented the most abundant identifiable taxon (5.5–11%).

Also similar to the metagenomes, the five most abundant organisms detected in the metatranscriptomes within sulfidic waters (except at 40 m) were related to SUP05, *R. magnifica*, *V. okutanii*, *Sulfurovum* and *D. autotrophicum*. We also identified several other taxa in the metatranscriptomes that were poorly represented in the metagenomes, some of which were closely related to members of hydrothermal vent communities. At 40 m, organisms related to the ε-proteobacterium *Arcobacter butzleri* RM4018 accounted for 2% of all RNA sequences and at 50 m the γ-proteobacterium *Colwellia psychrerythraea* 34H accounted for 3%. In deeper parts of the water column (60 and 80 m), organisms similar to the ε-proteobacteria *Sulfurimonas gotlandica GD1* (1.1–1.3%) and *Arcobacter nitrofigilis* DSM 7299 (0.9–1%) were detected. Although sequences similar to the anammox-planctomycete *Candidatus* Kuenenia stuttgartiensis have been found in high abundances in OMZ waters [14], [37], [40], they were relatively rare within our samples, never exceeding 0.7% of all DNA and 0.8% of all RNA sequences.

### Metabolic activity and functional diversity of the microbial community

#### General activity patterns

**Figure 5:**
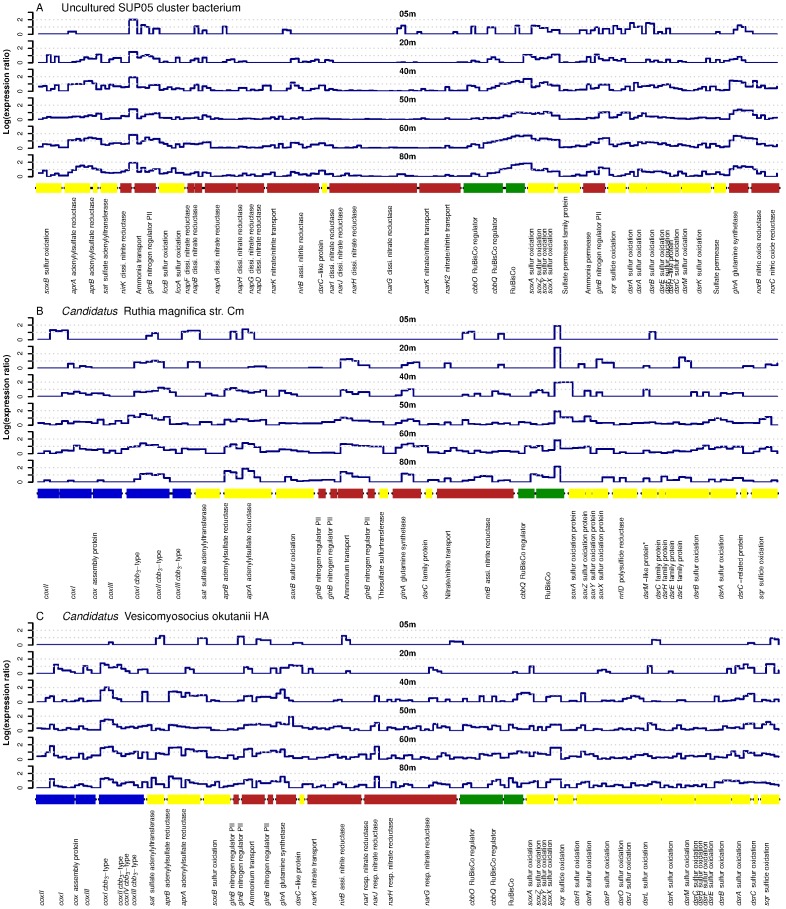
Vertical distribution of sequences recruited onto the genomes of three γ-proteobacterial sulfur oxidizers. Shown are selected genes encoding for enzymes involved in oxygen (blue), sulfur (yellow), nitrogen (red) and carbon metabolism (green) in the corresponding order of the genomes. The y-axis depicts the log of the expression-ratio, a measure for the selective enrichment of transcripts over the corresponding gene, normalized to the total pool of protein-coding sequences. A list of the start and end position of all genes and the full names of the corresponding enzymes are shown in Table S3. (A) Uncultured SUP05 cluster bacterium. (B) *Candidatus* Ruthia magnifica str. Cm. (C) *Candidatus* Vesicomyosocius okutanii HA. ^*^This DsrM-like protein has also high similarity to a *narG* respiratory nitrate reductase.

To assess the functional diversity of the microbial community in detail, we used three different approaches to investigate our sequence data. The BLAST-searches were supplemented by scans of our sequences with profile hidden Markov models of the ModEnzA Enzyme Commission (EC) groups [69] and of the Pfam protein families [70]. Furthermore, we recruited the DNA and RNA sequences onto the genomes of the five organisms most often recognized by our BLAST-searches, SUP05, *R. magnifica*, *V. okutanii*, *Sulfurovum* and *D. autotrophicum* [41], [64]–[67]. For these genome recruitments, we calculated the expression-ratio, a measure of the enrichment of selected transcripts over the corresponding genes, normalized to the total pool of all protein-coding sequences (Figure 5 and S4). In general, the genome recruitment plots showed high expression-ratios for ribosomal proteins, DNA and RNA polymerases, cell division proteins and transcription and translation factors indicating a growing microbial community (data not shown). Similarly, the collection of all abundant EC numbers also suggested an overall active microbial community (Figure S5). Sequences encoding for ubiquitous proteins related to general metabolic activity like DNA and RNA polymerases, DNA topoisomerases and adenosinetriphosphatases (a general ATP-binding and hydrolyzing motif in sequences which lack further specific functional information) were among the most abundant at all depths.

#### Hydrogen sulfide sources

H_2_S formation through microbial SO_4_^2−^ reduction commonly occurs in anoxic marine sediments, where it is considered to be the main heterotrophic process for the degradation of organic carbon [29]. The sedimentary flux has been shown to be the main source of H_2_S in the water column during sulfidic events in the Benguela Current upwelling system [30], [31]. However, at times water column SO_4_^2−^ reduction could also contribute significantly to the H_2_S accumulation in oceanic waters [34], [35], as demonstrated in the ∼2000 m thick anoxic water column in the Black Sea, where pelagic SO_4_^2−^ reduction rates ranged between 0.01–3.5 nmol l^−1^ d^−1^ [71]. Moreover, H_2_S formation from SO_4_^2−^ has been measured even in the presence of more favourable electron acceptors (NO_x_) in the OMZ waters off northern Chile after preincubations with H_2_S [14].

We investigated the sulfur cycling by identifying genes and transcripts indicative of specific metabolic functions related to sulfur transformation processes in our collection of sequences and used flux calculations to estimate the sedimentary source of H_2_S. *D. autotrophicum*, one of the most abundant organism we identified in our sequence dataset, is a metabolically versatile SO_4_^2−^ reducing marine δ-proteobacterium, which can completely oxidize organic carbon compounds to CO_2_, but is also capable of growing autotrophically on hydrogen (H_2_) [67]. The genome recruitment plots of *D. autotrophicum* (Figure S4B) show regions of the genome mostly related to energy metabolism and nutrient cycling. High expression-ratios for key sulfur metabolizing enzymes like the dissimilatory sulfite reductase (*dsrABD*), the adenylylsulfate reductase (*aprAB*) and the sulfate adenylyltransferase (*sat2*) suggest that *D. autotrophicum* could have been reducing SO_4_^2−^ and thus may have contributed to the formation of H_2_S at our sampling site. However, since many sulfur cycling proteins (e.g. *dsr* and *apr*) can function in both the oxidation and reduction of sulfur species [72], [73], other chemolithoautotrophic metabolic processes (e.g. the disproportionation of sulfur compounds resulting in the simultaneous formation of H_2_S and SO_4_^2−^) could have been catalyzed by these enzymes at the time of sampling [74]. The presence of large plumes of colloidal S^0^ (Figure S1) in the study area would have supported this chemolithoautotrophic reaction.

**Figure 6:**
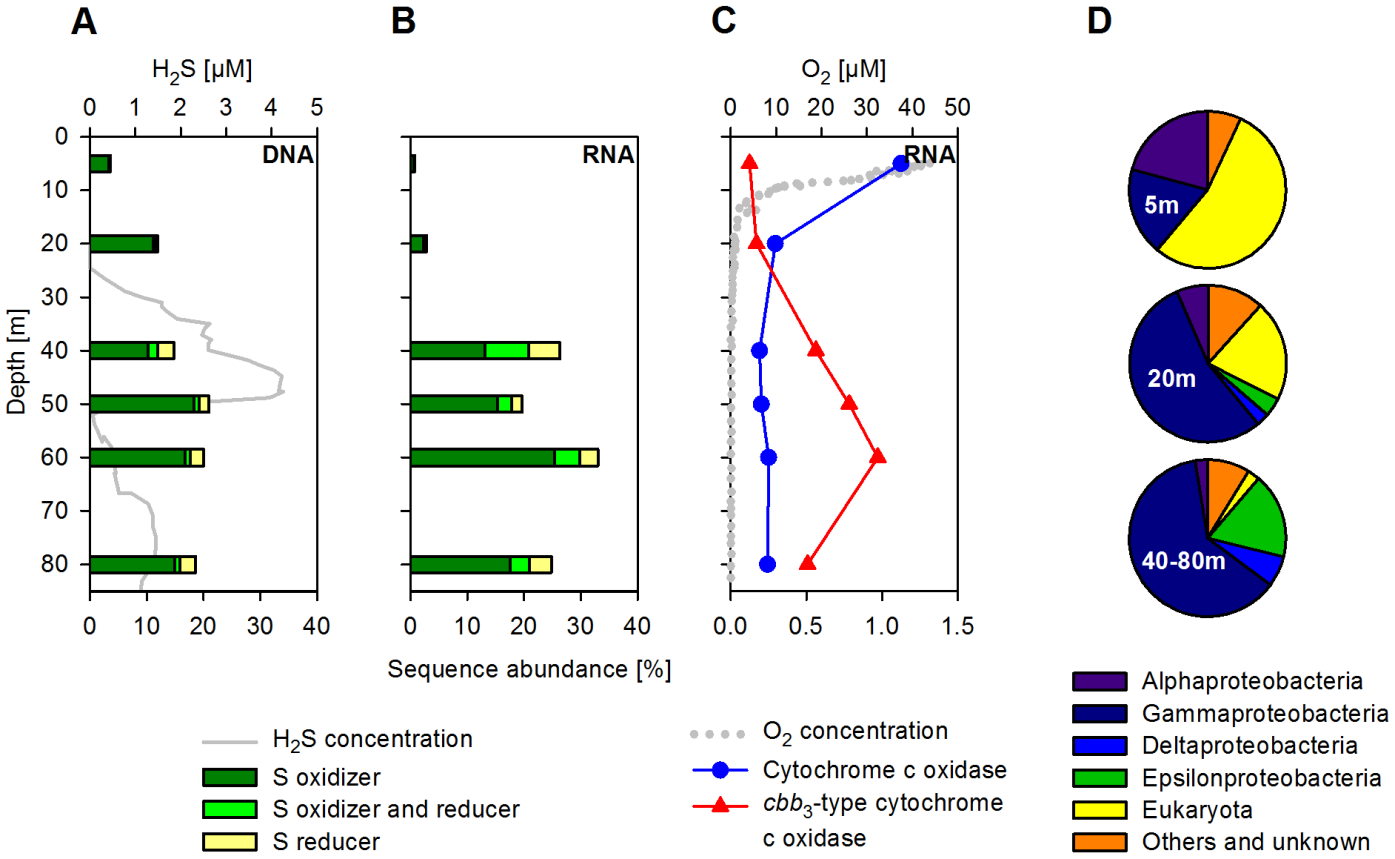
Vertical distribution of organisms involved in the sulfur cycle and abundance and taxonomic affiliation of transcripts encoding for cytochrome c oxidases. (A) H_2_S concentration and abundance of DNA sequences affiliated to organisms either capable of oxidizing or reducing inorganic sulfur species. Shown in percent of all DNA sequences (excluding rRNA genes) and summed according to their metabolic potentials. (B) Abundance of RNA sequences affiliated to organisms either capable of oxidizing or reducing inorganic sulfur species. Shown in percent of all RNA sequences (excluding rRNAs) and summed according to their metabolic potentials. (C) O_2_ concentrations and transcript abundance of the (low-affinity) cytochrome c oxidase and the (high-affinity) *cbb*_3_-type cytochrome c oxidase (both EC 1.9.3.1). Shown in percent of all protein-coding RNA sequences. (D) Phylogenetic affiliation of the transcripts encoding for both types of the cytochrome c oxidase.

A numerical compilation of all sequences affiliated to known SO_4_^2−^ reducers and sulfur oxidizers is shown in Figure 6 [14], [72], [73]. Although the abundance of SO_4_^2−^ reducing organisms throughout sulfidic waters was not correlated with the H_2_S concentrations, it is likely that they contributed to SO_4_^2−^ reduction and H_2_S formation in the water column at the time of sampling. However, assuming the maximum SO_4_^2−^ reduction rates (1.3–12 nmol l^−1^ d^−1^) measured in OMZ waters off northern Chile [14], it would take more than one year (in the absence of any oxidation) to accumulate the H_2_S concentrations reported in our study. In contrast, SO_4_^2−^ reduction in sediments underlying the eastern tropical South Pacific OMZ is generally very high (10–30 mmol m^−2^ d^−1^) [75] and thus the most likely source of the H_2_S we measured in the water column.

Assuming steady state conditions, we used the density structure and the H_2_S concentration gradient (Figure 2A) in the bottom water to estimate a turbulent diffusion of ∼10^−4^ m^2^ s^−1^ and, subsequently, a sedimentary efflux of ∼2 mmol H_2_S m^−2^ d^−1^. This is well within the estimates based on sedimentary flux calculations and SO_4_^2−^ reduction rate measurements (1–11 mmol m^−2^ d^−1^) from sediments directly underlying sulfidic events [76]. Moreover, the repeated observations of the H_2_S maxima in bottom waters along the transect (Figure 1C) point towards the sediment as the main H_2_S source.

We observed a second distinct H_2_S maximum in the water column at ∼48 m with no direct contact to the sediment. However, the salinity and the corresponding PO_4_^3−^ and NH_4_^+^ concentrations (Figure 2C and 2E) indicated that the upper H_2_S layer was most probably created by lateral advection of nearby bottom waters that had recently been in contact with sulfidic sediments rather than by production of H_2_S within the water column.

#### Sulfur oxidation

The largest functional group of microorganisms detected in sulfidic waters were γ–proteobacterial sulfur oxidizers. Figure 5 shows the expression-ratio for selected genome regions of the three most abundant GSO-representatives. The recruitment of the sequences onto separate genomes supported the presence of at least three distinct taxa within the GSO-community (similar to SUP05, *R. magnifica* and *V. okutanii*) at our study site, which were actively growing and metabolizing. Figure S4A further depicts a genome recruitment plot for ε-proteobacterial *Sulfurovum*. The recruitment of sequences onto the genome of *Sulfurovum* was much less extensive than for the GSO-group, and showed low coverage especially in the oxic and anoxic depths (5 and 20 m).

A large number of transcripts were recruited onto genes (if present in the genomes) of the reverse dissimilatory sulfite reduction (*dsr* - oxidation of intracellular S^0^) and the periplasmic sulfur oxidation (*sox* - S_2_O_3_^2−^ oxidation) pathways, both encoding for enzymes involved in the oxidation of reduced sulfur compounds. Additionally, we found high expression-ratios for the sulfide∶quinone oxidoreductase (*sqr* - H_2_S oxidation to S^0^), the adenylylsulfate reductase (*apr*) and the sulfate adenylyltransferase (*sat* - latter both SO_3_^2−^ oxidation to SO_4_^2−^). The abundance and coverage of transcripts matching these clusters for the GSO-group and *Sulfurovum* increased generally with depth, delivering the highest expression-ratios of genes encoding for sulfur oxidizing proteins within sulfidic waters. For *Sulfurovum*, which is thought to be capable of both sulfur oxidation and SO_4_^2−^ reduction [66], [77], [78], the transcript coverage for its *sox*- and *sqr*-genes suggested that it was likely acting as a sulfur oxidizer at the time of sampling.

*R. magnifica*, *V. okutanii* and *Sulfurovum* genomes harbour genes for different cytochrome c oxidases (*cox*) which are used in oxic respiration and are absent in the currently annotated version of the SUP05 genome [41], [64]–[66]. Of the transcripts recruiting to the *R. magnifica* genome, the cytochrome c oxidase was among the most abundant in the oxic surface. In anoxic and sulfidic waters, however, expression patterns changed and transcripts for a *cbb*_3_-type cytochrome c oxidase dominated instead. Transcripts for the *cbb*_3_-type cytochrome c oxidase recruiting to the genomes of *V. okutanii* and *Sulfurovum* were also among the most highly expressed in sulfidic waters.

The *cbb*_3_-type cytochrome c oxidase is thought to be involved in a specialized microaerobic respiration. This enzyme has an extremely high affinity to O_2_, with a *K_M_* value as low as 7 nM, allowing certain proteobacteria to colonize oxygen-limited or even presumed anoxic environments [79], [80], well below the detection limit of the microsensor (0.5–1 µM) and the STOX sensor (∼50 nM) we used in this study. Furthermore, it was shown that *Escherichia coli* cultures actively grew and respired O_2_ even below the detection limit of a highly sensitive STOX sensor (≤3 nM), probably using a high-affinity cytochrome *bd* oxidase [81].

A clear separation of the cytochrome c oxidase expression was visible between the oxic and the anoxic/sulfidic zone, directly reflecting the availability of O_2_ (Figure 6C). In oxic surface waters, the low-affinity cytochrome c oxidase was dominant and assigned mainly to eukaryotic sequences, as well as to diverse bacterial groups belonging to α- and γ-proteobacteria (Figure 6D). In contrast, in sulfidic waters where the high-affinity *cbb*_3_-type cytochrome c oxidase prevailed, as much as 80% of the transcripts could be assigned to either γ- and ε-proteobacteria. For the *R. magnifica*-like organism, which possesses both types of cytochrome oxidases, the switch in the expression from the low-affinity type in oxic surface waters (5 m) to the high-affinity type in anoxic and sulfidic waters (20–80 m) can be observed in the genome recruitment plots (Figure 5B).

Despite the presence of H_2_S, the oxygen microsensor showed trace amounts of O_2_ (<1 µM) down to 40 m water depth during the downcast, which might be an artefact from water advection caused by the CTD rosette (see section ‘description of the sampling site’). Assuming that the measured O_2_ concentrations were not an artifact, we calculated a vertical down flux of 0.07 mmol O_2_ m^−2^ d^−1^ between 17 and 27 m. Although this O_2_ flux could only account for the oxidation of ∼6% of the sedimentary H_2_S flux of 2 mmol m^−2^ d^−1^, it could partly sustain microaerobic activity in the sulfidic waters as suggested by the presence of the *cbb*_3_-type cytochrome c oxidase transcripts. Lateral advection of oxic waters and water exchange induced by internal waves may also have supported microaerobic activity at O_2_ concentrations that would remain below the detection limit of our STOX sensor (∼50 nM). However, mixing due to internal waves would have influenced only the upper part of the water column and the widespread anoxic conditions below 30 m gave little indication of lateral advection of oxic waters from 30–100 m, where the *cbb*_3_-type cytochrome c oxidase was nevertheless strongly expressed. Either, the O_2_ was consumed shortly before our sampling campaign or advective transport of oxic waters supplied O_2_ in concentrations below the detection limit of our STOX sensor. An alternative explanation for the high *cbb*_3_-type cytochrome c oxidase expression in the sulfidic waters could also be the use of nitric oxide (NO) as an alternate substrate instead of O_2_, as hypothesized for a γ-proteobacterial species [82] and supported by structural similarities of *cbb*_3_-type cytochrome c oxidases to bacterial nitric oxide reductases [83]. Although we did not measure NO in our study, a high expression-ratio of the genes encoding for nitric oxide reductases (*norBC*) were found in the genome recruitments for SUP05 (these genes are absent from currently available versions of the *R. magnifica* and *V. okutanii* genomes) in sulfidic depths, and at 60 m also for *Sulfurovum*. Furthermore, transcripts for the dissimilatory nitrite reductase (*nirK*) were highly expressed in SUP05-like organisms throughout the water column. Since this enzyme reduces NO_2_^−^ to NO, minor concentrations of NO might have been available for respiration processes linked to sulfur oxidation.

Alternatively to O_2_ (and NO) utilization, SUP05, *V. okutanii* and *Sulfurovum* and several other ε-proteobacteria [84], [85] are also capable of coupling the oxidation of H_2_S to the reduction of NO_x_. Low but persistent concentrations of NO_x_ were measurable at most depths down to 60 m, and nitrate as well as nitrite reductase (*nar-*, *nap- and nir-*genes) transcripts were expressed in all anoxic and sulfidic depths (Figure 5 and S4). The genome recruitment plots for SUP05 (the genome contains genes from all three clusters) show that *nap*- and especially *nir*-genes were expressed, while relatives of *V. okutanii* and *Sulfurovum* showed high expression-ratios mostly for *nar*- and *nap*-genes, respectively. In contrast, most of the genes mentioned above are absent from the currently available version of the *R. magnifica* genome and it is thought that *R. magnifica* reduces NO_x_ for assimilatory reasons only [65].

**Figure 7:**
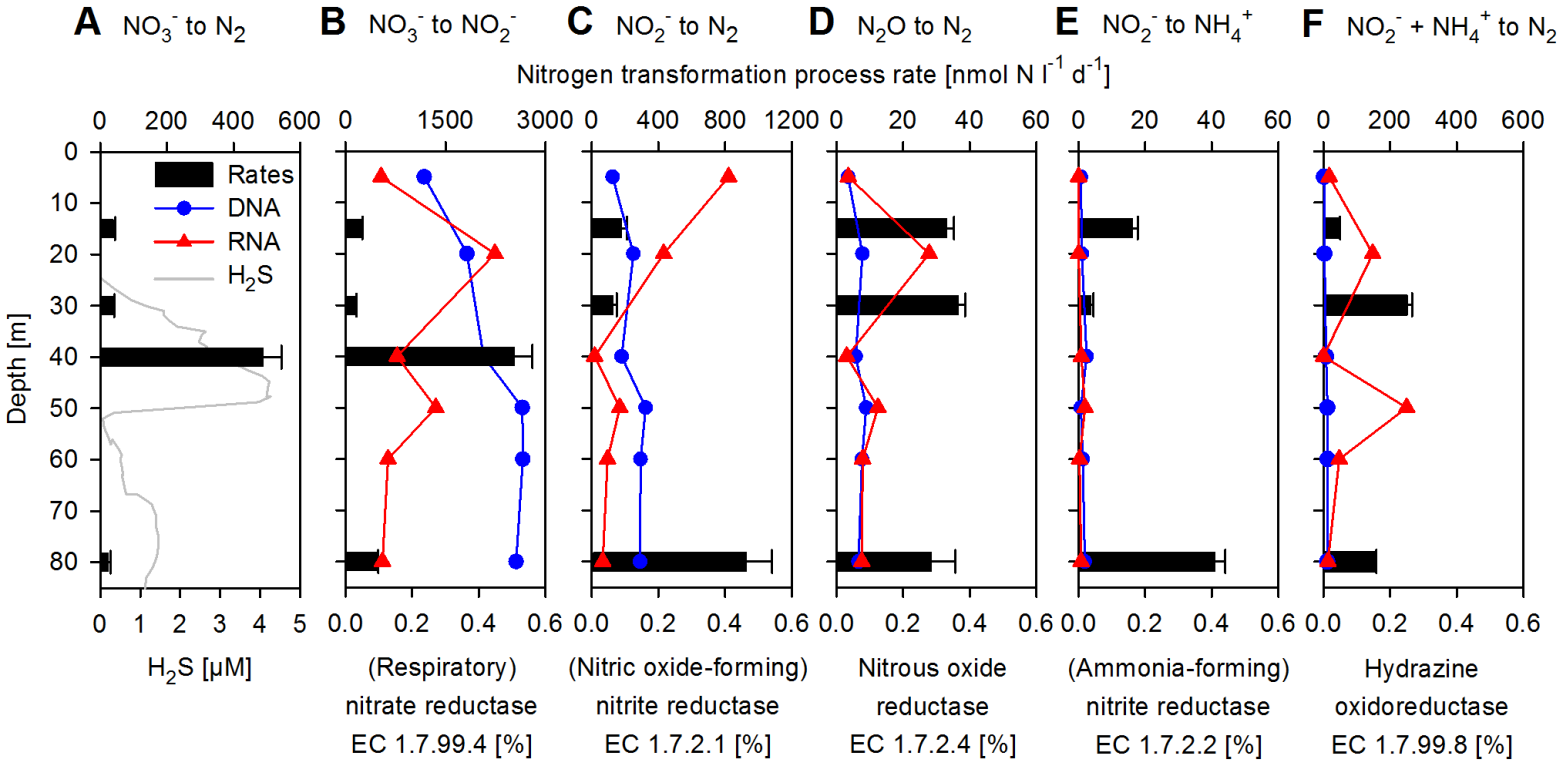
Vertical distribution of nitrogen transformation process rates and abundances of sequences encoding for involved enzymes. Shown in percent of all protein-coding DNA and RNA sequences, respectively. (A) NO_3_^−^ reduction to N_2_ (denitrification). (B) NO_3_^−^ reduction to NO_2_^−^, respiratory nitrate reductase (EC 1.7.99.4). (C) NO_2_^−^ reduction to N_2_, (NO forming) nitrite reductase (EC 1.7.2.1). (D) N_2_O reduction to N_2_, nitrous-oxide reductase (EC 1.7.2.4). (E) NO_2_^−^ reduction to NH_4_^+^ (DNRA), (NH_4_^+^ forming) nitrite reductase (EC 1.7.2.2). (F) NO_2_^−^+NH_4_^+^ to N_2_ (anammox, based on the sole addition of NO_2_^−^), hydrazine oxidoreductase (EC 1.7.99.8). Please note that at 40 m only NO_3_^−^ reduction to N_2_ (A) and NO_3_^−^ reduction to NO_2_^−^ (B) were measured.

We could not calculate fluxes from the trace amounts of NO_x_ below 30 m. However, at the upper boundary of the second H_2_S peak (25–29 m) the NO_2_^−^ and H_2_S profiles overlap. The ratio of the opposing concentration gradients of NO_2_^−^ (−0.53 µM m^−1^) and H_2_S (0.22 µM m^−1^) between 25 and 29 m depth indicates that the NO_2_^−^ flux was sufficient to oxidize the upward flux of H_2_S. A diffusion coefficient of ∼2×10^−5^ m^2^ s^−1^ can be estimated from the density gradient at this depth, resulting in an upward flux of 0.38 mmol H_2_S m^−2^ d^−1^ and an average oxidation rate of ∼100 nmol H_2_S l^−1^ d^−1^ within the ∼4 m thick overlapping layer. This is in the same range as the experimentally measured reduction of NO_2_^−^ to N_2_ (126 nmol N l^−1^ d^−1^) at 30 m depth (see section ‘nitrogen cycling’ and Figure 7), suggesting that H_2_S oxidation was carried out partly via sulfur-driven autotrophic denitrification. The removal rate of NO_2_^−^ calculated from the downward flux of NO_2_^−^ was 230 nmol N l^−1^ d^−1^ and matched the experimentally measured NO_2_^−^ removal of 255 nmol N l^−1^ d^−1^ from combined denitrification, anammox and dissimilatory nitrate reduction to ammonia (DNRA).

Although our results suggest that H_2_S was oxidized by both microaerobic activity and sulfur-driven autotrophic denitrification well below the oxic zone (>1 µM O_2_), our flux calculations indicate that the larger part of the H_2_S was probably oxidized anaerobically with NO_x_.

#### Nitrogen cycling

To shed light on the nitrogen cycling carried out by the microbial community, we measured potential rates of various nitrogen transformation processes using ^15^NO_3_^−^, ^15^NO_2_^−^, ^15^N_2_O or ^15^NH_4_^+^ incubations and compared them with corresponding abundances of functional genes and transcripts (the sum of BLAST-hits, EC number- and Pfam-assignments) involved in their turnover (Figure 7). Reduction of NO_3_^−^ to NO_2_^−^ was active throughout the anoxic and sulfidic zones, with the highest rates measured at 40 m (2500 nmol N l^−1^ d^−1^; Figure 7B). In comparison, the much lower rate measured at 30 m (150 nmol N l^−1^ d^−1^) might have been caused by limitations in reductants, as suggested by the much lower H_2_S concentrations at that depth. The transcript abundance for respiratory nitrate reductase (EC 1.7.99.4) peaked in the anoxic zone at 20 m (0.35% of all protein-coding sequences) and then dropped within the sulfidic waters following the decrease in NO_3_^−^ concentrations. Gene abundances however, increased with depth (∼0.5%) and it is possible that the addition of NO_3_^−^ necessary for rate measurements stimulated a fast response of the microbial community yielding the actual potential for NO_3_^−^ reduction to NO_2_^−^ rather than the *in situ* rate. The majority of the genes and transcripts encoding for respiratory nitrate reductase at 5 and 20 m were similar to *K. stuttgartiensis*, while transcripts in sulfidic waters belonged to diverse groups of α-, β-, γ-, δ- and ε-proteobacteria, indicating a clear taxonomic separation with depth.

Measured rates for the subsequent steps in denitrification, the reduction of NO_2_^−^ to N_2_ were highest close to bottom waters at 80 m (900 nmol N l^−1^ d^−1^; Figure 7C), whereas the transcript abundance for NO-forming cd-cytochrome nitrite reductase (EC 1.7.2.1) was highest in the oxic and anoxic zones at 5 and 20 m (0.4% and 0.2%), mirroring the availability of NO_2_^−^. The majority of the transcripts at 5 and 20 m were similar to the gene from *N. maritimus*, while those recovered from sulfidic depths were affiliated with diverse groups of proteobacteria. We also found high expression of ammonia monooxygenase (EC 1.14.99.39) transcripts related to *N. maritimus* (1.5%, data not shown) in the oxic surface (5 m), which may be partly responsible for the high NO_2_^−^ concentrations in the surface waters. Some of the NO_2_^−^ reduction (N_2_ production) could be attributed to anammox activity, with the highest rates measured at 30 m (250 nmol N l^−1^ d^−1^ based on ^15^NO_2_^−^ addition, Figure 7F and 96 nmol N l^−1^ d^−1^ based on ^15^NH_4_^+^ addition, data not shown). At 80 m anammox activity could only be detected by ^15^NO_2_^−^ addition (152 nmol N l^−1^ d^−1^); an incubation with added ^15^NH_4_ did not stimulate any N_2_ production, most likely due to limitations in NO_x_. The abundance of genes encoding for hydrazine oxidoreductase (EC 1.7.99.8) was generally very low at all depths (less than 0.015%; Figure 7F) while transcripts, mostly annotated as similar to *K. stuttgartiensis*, peaked in abundance at 20 and at 50 m (0.15 and 0.25%, respectively). The 50 m transcript maximum of hydrazine oxidoreductase (we did not measure rates at this depth) is in good agreement with a small peak in NO_3_^−^ concentrations (to 0.7 µM) and a minor dip in NH_4_^+^ concentrations (Figure 2C). The transcript maximum might also be influenced by the much lower H_2_S concentrations at this depth, as anammox activity was shown to be inhibited by H_2_S [86].

N_2_O is an intermediate in denitrification (NO_x_ to N_2_) and we measured the reduction of N_2_O to N_2_, which turned out to be of smaller magnitude (30 nmol N l^−1^ d^−1^) than the NO_2_^−^ reduction to N_2_ (Figure 7D). The N_2_O concentrations, which ranged between 20 and 40 nM from surface to ∼80 m, dropped below detection limit at 80 m (Figure 2B), indicating either a complete reduction of N_2_O as previously observed for this area [87] or a lack of production due to the limitation in NO_x_. Gene and transcript abundance for nitrous oxide reductase (EC 1.7.2.4) were highest in anoxic waters (20 m) reaching 0.3% and comparable in magnitude to the abundances of sequences encoding for nitrate and nitrite reductases from the same depths.

We also conducted rate measurements of complete denitrification (NO_3_^−^ to N_2_; Figure 7A). The highest rate (490 nmol N l^−1^ d^−1^) was found at 40 m within the first H_2_S maximum. Much lower rates were observed for the other depths (26–41 nmol N l^−1^ d^−1^), which might be attributed to incomplete denitrification (e.g. NO_3_^−^ reduction to NO_2_^−^, NO or N_2_O) and potentially also to limitations in concentrations of the reductant (H_2_S).

Rates for DNRA were the lowest of the nitrogen transformations processes we measured, not exceeding 40 nmol N l^−1^ d^−1^ (Figure 7E). Gene abundance for cytochrome c nitrite reductase (EC 1.7.2.2) was also lower than genes implicated in the other nitrogen transformation processes. Except for 50 m depth, the transcripts for this gene were even rarer than the gene abundances (less than 0.01%). This suggests only a minor role of DNRA in the microbial community metabolism at the time of sampling.

Although rate measurements do not provide information on the phylogenetic affiliation of organisms carrying out the nitrogen transformations, the predominance of sulfur oxidizing proteobacteria found in our sequence data suggests that the sulfur-driven autotrophic denitrification was most likely the dominant pathway for N-loss during our sampling campaign.

#### Carbon assimilation

**Figure 8:**
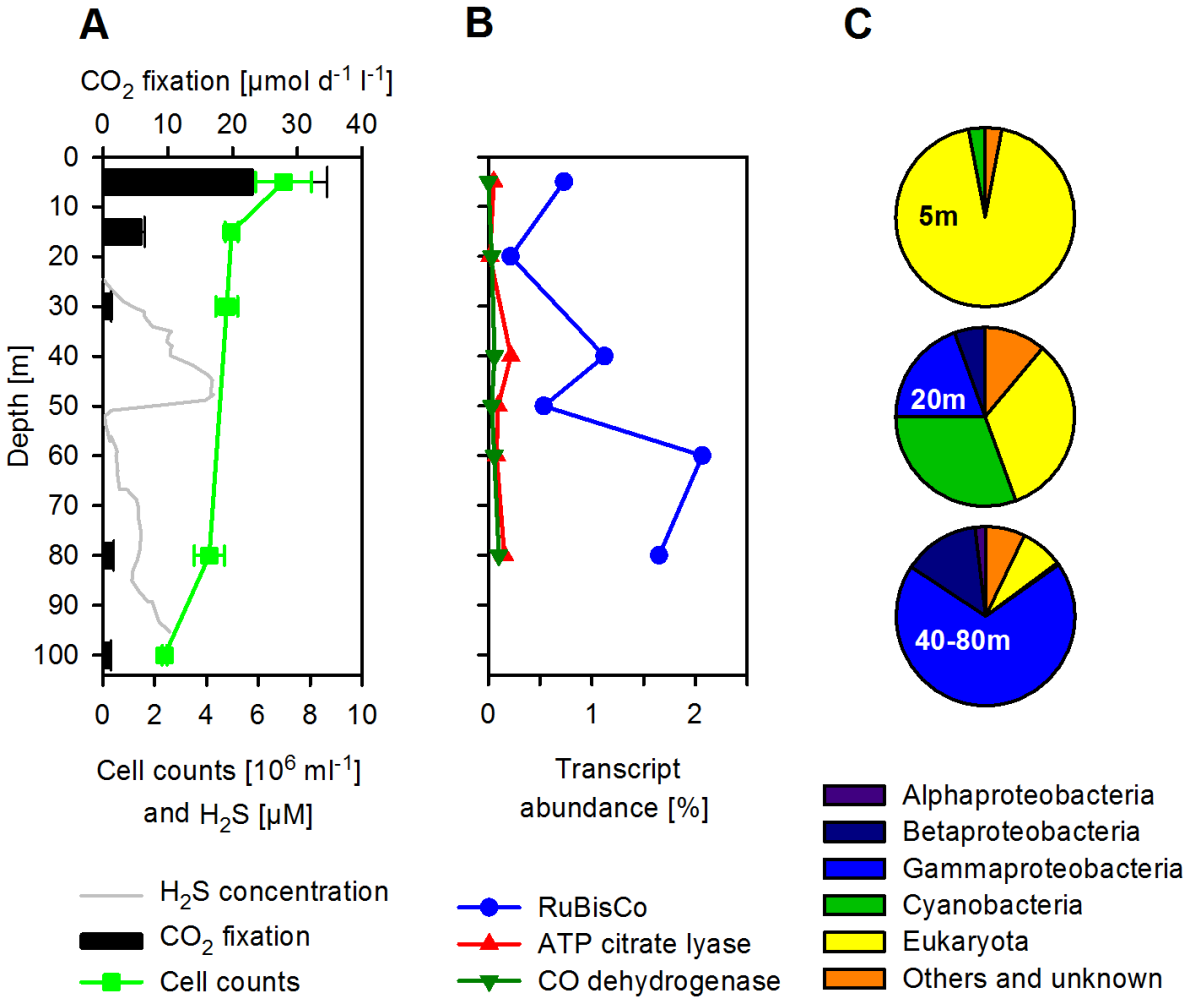
Vertical distribution of CO2 fixation rates, microbial cell counts and abundance of transcript encoding for key carbon-fixing enzymes. (A) CO_2_ fixation rates and total microbial cell counts. (B) Abundances of transcripts encoding for carbon-fixing enzymes (in percent of all protein-coding RNA sequences): Ribulose-1,5-bisphosphate carboxylase oxygenase (RuBisCo, EC 4.1.1.39, Calvin-Benson-Bassham cycle), ATP citrate lyase (EC 2.3.3.8, Arnon-Buchanan cycle) and CO-dehydrogenase (EC 1.2.99.2, Wood-Ljungdahl pathway). (C) Phylogenetic affiliation of the transcripts encoding for RuBisCo.

Eastern Boundary Upwelling Systems are characterized by high primary productivity due to photosynthetic (photoautotrophic) growth in surface waters, which in turn stimulates heterotrophic respiration processes in underlying waters. However, autotrophic lifestyles have also been found in these underlying waters, e.g. by organisms responsible for nitrification [28], [88], anammox [22]–[24], [26] and sulfur-driven autotrophic denitrification [31], [44], [84]. To investigate the magnitude of inorganic carbon assimilation of the microbial community, we conducted rate measurements at selected depths with ^13^C-bicarbonate incubations and compared them to the relative abundance of transcripts encoding for key carbon-fixing enzymes (Figure 8). High abundances of transcripts for ribulose-bisphosphate carboxylase/oxygenase (RuBisCo, EC 4.1.1.39) were found at all depths, but especially in sulfidic waters. At 40, 60 and 80 m transcripts encoding for RuBisCo were the most abundant to be identified in the metatranscriptomes (1.1, 2.1 and 1.6% of all protein-coding sequences, respectively; Figure 8B and Figure S5). The phylogenetic diversity of the RuBisCo transcripts varied throughout the water column. Transcripts from photosynthetic organisms such as algae and diatoms (e.g. similar to *Heterosigma akashiwo*, *Odontella sinensis* and *Thalassiosira* spp.) as well as cyanobacteria dominated the 5 and 20 m samples. In the sulfidic zone (40–80 m), β- and especially γ-proteobacterial transcripts were most abundant (Figure 8C). Approximately 25% of all RuBisCo-transcripts were most similar to a bacterial artificial chromosome-clone of unknown bacterium 560, which also appears to belong to a SUP05 genome [89]. Altogether, transcripts from γ-proteobacteria contributed about 70% of all RuBisCo transcripts in sulfidic waters.

While the high proportions of RuBisCo transcripts were indicative of an active Calvin-Benson-Bassham cycle, other CO_2_ fixation pathways were also represented by the presence of transcripts for key enzymes of the Arnon-Buchanan cycle (ATP citrate lyase, EC 2.3.3.8) and the Wood-Ljungdahl pathway (CO-dehydrogenase, EC 1.2.99.2). Transcripts encoding for these two enzymes accounted for up to 0.3% of all protein-coding sequences at 40 m depth (Figure 8B) and were also recruited onto the *Sulfurovum* and *D. autotrophicum* genomes, respectively (Figure S4).

CO_2_ fixation rates measured at selected depths with ^13^C-bicarbonate incubations (Figure 8A) were highest in the nutrient-rich oxic surface waters (23 µmol C l^−1^ d^−1^), reflecting the dominance of large-sized eukaryotic phytoplankton. However, dark incubations with ^13^C-bicarbonate yielded chemolithoautotrophic CO_2_ fixation rates ranging from 0.9 to 1.4 µmol C l^−1^ d^−1^ at depths of 30, 80 and 100 m. These measured rates are comparable to chemolithoautotrophic activity found in the redoxclines and the sulfidic zones of the Baltic and the Black Sea [42], [43], [90]–[93]. In comparison, carbon assimilation rates of 0.3 µmol C l^−1^ d^−1^ by heterotrophic bacteria, after the addition of ^13^C-glucose (data not shown), was only about one third when compared with the chemolithoautotrophic CO_2_ fixation. Based on total microbial cell counts, each cell fixed ∼0.3 fmol C d^−1^, a magnitude that was also observed in the sulfidic zone of the Baltic Sea [92].

Integrating the CO_2_ fixation rates over the predicted predominantly photic zone (0–20 m) we estimated ∼288 mmol C m^−2^ d^−1^ being fixed. This is in good agreement with the highest primary production rates modelled for the study area during the same period of time (250 mmol C m^−2^ d^−1^) [25]. On the other hand, the integrated light-independent CO_2_ fixation rates in the predicted predominantly aphotic zone (20–100 m) reached ∼96 mmol C m^−2^ d^−1^. Consequently, ∼25% of the total CO_2_ fixation at our study site was carried out by chemolithoautotrophic microorganisms. This is similar to the dark CO_2_ fixation in the redoxcline of the Baltic Sea, which was shown to contribute ∼30% of the surface productivity and to the dark CO_2_ fixation rates as reported for Chilean waters, which accounted for an average of ∼20% of total CO_2_ fixation [88], [94]. The chemolithoautotrophic CO_2_ fixation at our study site could represent as much as 33–53% of the estimated average CO_2_ fixation rate per square meter for the Humboldt Current System (182–290 mmol C m^−2^ d^−1^) [2], [4]. Assuming the measured rates were maintained throughout the entire sulfidic plume (∼5500 km^2^), the CO_2_ fixed through chemolithoautotrophy would have been ∼6.3×10^3^ tons C per day. In comparison, in this area an estimate of the total primary production by remote sensing is ∼5.5×10^5^ tons C per day [2]. Consequently, the chemolithoautotrophic activity during this sulfidic event would have contributed ∼1.2% of total CO_2_ fixed in the Humboldt Current System off the Peruvian coast at the given time. This is intriguing, since the Humboldt Current System is one of the most productive marine systems world-wide and supports the production of more fish per unit area than anywhere else in the world [2]–[4], [95]. Moreover, since the sulfidic plume may have been considerably larger than the extent we recorded and was either recurrent or prevailed for several months, the chemolithoautotrophic growth is significant in terms of carbon retention. Considering that only 15–30% of the photosynthetic surface production is actually exported to OMZ waters [25] the chemolithoautotrophic growth may act as an important, but up to now neglected factor promoting SO_4_^2−^ reduction and thus stabilizing sulfidic conditions in OMZ waters.

### Conclusions

The Eastern Boundary Upwelling System off the Peruvian coast is one of the world's most productive oceanic regions and comprises one of the largest OMZs world-wide [2], [4], [11]. We reported here the detection of a sulfidic plume within continental shelf waters of the Peruvian OMZ in January 2009. The sulfidic plume covered an area >5500 km^2^ and contained ∼2.2×10^4^ tons of toxic H_2_S, representing with ∼440 km^3^ the largest sulfidic plume ever observed in oceanic waters and the first time that H_2_S was measured in Peruvian OMZ waters.

The microbial community was largely dominated by several distinct γ- and ε-proteobacteria related to SUP05, *R. magnifica*, *V. okutanii* and *Sulfurovum*, which transcribed a broad range of genes involved in sulfur (H_2_S, S_2_O_3_^2−^, S^0^ and SO_3_^2−^) oxidation. Our data suggested that these sulfur oxidizing proteobacteria probably utilized several different oxidants ranging from O_2_, NO_3_^−^, NO_2_^−^, NO to N_2_O to oxidize the H_2_S well below the oxic surface. While sequences related to SUP05 indicated that genes involved in the reduction of NO_x_ and NO were being expressed, *R. magnifica*-, *V. okutanii*- and *Sulfurovum*-like transcripts related to a microaerophilic *cbb*_3_-type cytochrome c oxidase also pointed to the use of O_2_ for H_2_S oxidation.

High-throughput sequencing data further showed a high abundance and transcriptional activity of δ-proteobacterial SO_4_^2−^ reducing *D. autotrophicum*. Transcripts recruiting to the SO_4_^2−^ reduction genes of *D. autotrophicum* were found suggesting that SO_4_^2−^ reduction may have occurred in the water column. However, our flux calculation indicated that the main source for H_2_S in the water column was sedimentary SO_4_^2−^ reduction. Given the fact that many sulfur cycling enzymes can both oxidize and reduce sulfur species [72], [73] and the presence of colloidal S^0^ plumes within the sampling area as observed with remote satellite sensing, the disproportionation of sulfur compounds could also be a way of energy acquisition for some of the proposed SO_4_^2−^ reducers [74].

High CO_2_ fixation rates were measured in photic surface waters (5.8–23 µmol C l^−1^ d^−1^), but also in the dark sulfidic zone, ranging from 0.9 to 1.4 µmol C l^−1^ d^−1^. Many identified microorganisms, both sulfur oxidizers and SO_4_^2−^ reducers were expressing transcripts encoding for key carbon-fixing enzymes. The light-independent, chemolithoautotrophic CO_2_ fixation is similar to observations made in permanently stratified systems like the Baltic and the Black Sea [42], [43], [90]–[93]. If the rates prevailed throughout the entire sulfidic zone, they would have represented as much as ∼30% of the photoautotrophic CO_2_ fixation at that site and would have been of similar magnitude as the photosynthetic surface production that is exported to OMZ waters [25].

The presence of colloidal S^0^ plumes in the study area again in May 2009 indicated that sulfidic waters in the OMZ off Peru might be more frequent and persistent than originally thought. Although the frequency and duration of H_2_S accumulations cannot be estimated from direct observations, their occurrence might increase in future resulting from eutrophication and global warming [27], [31], [35]. In addition, the carbon retention due to the chemolithoautotrophic activity presented in this study may enhance SO_4_^2−^ reduction and consequently H_2_S formation and thus could act as an important mechanism to stabilize sulfidic conditions in OMZ waters, potentially reducing the liveable habitat of many higher organisms.

## Materials and Methods

### Sample Collection

All waters samples were collected during RV Meteor cruise M77/3 (December 27^th^, 2008 to January 24^th^, 2009) on the Peruvian continental shelf. Station 19 (January 9^th^, 2009; 12°21.88′S, 77°0.00′W), located approximately 15 km off the coast of Lima was selected for detailed analysis. During the upcast, water was pumped from depth directly on board using a pump-conductivity-temperature-depth (pump-CTD) water sampler. We monitored density as well as O_2_ and H_2_S to account for internal waves and moved the pump-CTD accordingly.

Samples for nucleic acid extraction were filled (oxygen-free) in 4.5 l polycarbonate bottles. For each sample, 1.5–2 l of the water was prefiltered through 10 µm pore size filters (Whatman Nuclepore Track-Etch) and then collected upon 0.22 µm pore size filters (Millipore Durapore Membrane) using a vacuum pump. Less than 18 minutes elapsed from the time when the water was pumped on board until the filters were put in microcentrifuge reaction tubes and flash frozen in liquid nitrogen. Samples for incubation experiments and nutrient analysis were taken in parallel (see description below).

### RNA extraction and cDNA synthesis

DNA and RNA were extracted using the DNA/RNA-Allprep kit (Qiagen) with minor modifications in the protocol for the lyses step: The frozen filters were crushed using a disposable pestle and incubated with 200 µl lysozyme (10 µg/µl) and 1 mM EDTA at ambient temperatures for 5 minutes. Subsequently, 40 µl of Proteinase K (10 µg/µl) were added, followed by another incubation of 5 minutes at ambient temperatures. After adding 500 µl buffer RLT-Plus (containing 10 µl/ml β-mercaptoethanol) the manufacturer's instructions were followed. DNA was eluted in 150 µl elution buffer; RNA in 50 µl nuclease-free water, followed by a subsequent step of DNA digestion (Turbo DNA-free kit, Ambion). Prokaryotic rRNA was removed with *mRNA only* prokaryotic mRNA isolation kit (Epicentre). Further depletion of bacterial rRNA was achieved by using the Ambion Microb*Express* kit (see also section ‘sequencing statistics’). Cleaned and rRNA-depleted mRNA was subjected to an *in vitro* transcription (amplification) step using Ambion Message*Amp*. Finally, cDNA was synthesised using the Invitrogen superscript III cDNA synthesis kit with random hexameric primers (Qiagen). Throughout the procedure all DNA and RNA samples were subsequently quantified with a NanoDrop spectrophotometer and checked for degradation with a BioRad Experion Automated Electrophoresis System. Leftover reactants and reagents were removed using the PCR Mini Elute Kit (Qiagen). DNA and RNA was stored at −80°C until pyrosequencing. Throughout the whole procedure nuclease-free plastic consumables and nuclease-free water and reagents were used to hinder any possible degradation of DNA or RNA.

### Sequencing

For both DNA and RNA (cDNA), 50 µl were sequenced with the GS-FLX (Roche) pyrosequencer at the Institute of Clinical Molecular Biology in Kiel. Each sample was loaded on one quarter of a PicoTiter plate (except the 5 m RNA sample, which was loaded on two quarters of a PicoTiter plate). This resulted in 1,888,768 (DNA) and 1,560,959 (RNA) sequences with an average length of 392 base pairs, accounting for 757,439,211 and 599,103,110 base pairs of sequence information, respectively (Table S1).

### Sequence annotation pipeline

The sequence data was organized and analyzed with the Meta2Pro annotation pipeline [96]. All raw sequences were clustered using Cd-hit [97] with a sequence identity threshold of 98% and a word length of 8, delivering about 1,581,637 (DNA) and 592,711 (RNA) cluster representative sequences in total. The rRNA genes and rRNAs in these cluster representatives were identified by a BLASTn-search [98] against the SILVA database [99] including both prokaryotic and eukaryotic small and large ribosomal subunit sequences with a bit score cut off of 86. The bit score cut off of 86 as described earlier [100] was validated using a simulation exercise. All sequences deposited in the SILVA database (477,749 sequences) were randomly fragmented into one million sequences to simulate a pyrosequencing run. The generated fragments had a normal length distribution with mean and standard deviation values similar to those from our own dataset (mean: 420 base pairs and standard deviation: 150 base pairs). This simulated dataset was Cd-hit-clustered (as described above) and BLAST-searches against the SILVA database itself were carried out. All queries which hit themselves in the database (e.g. the sequence of origin of the query) or hit a sequence belonging to the same taxonomic lineage (allowing a mismatch of up to two taxonomic levels) were considered true positives, while those fragments that hit sequences from other taxonomic lineages were false positives. The bit score distributions for the true and false positives were binned and a threshold sweep was used to calculate the sensitivity and specificity value at each bit score threshold. A bit score value of 86 in the resulting plot of the sensitivity and specificity distributions for this threshold sweep gave a specificity of 99.35% and a sensitivity of 99.85% respectively. Hence, this cut off was used for all further analysis of the sequences against the SILVA database and also in MEGAN to make taxonomic assignments (using a minimum support of 5 and a 10% score range for its LCA algorithm) [101]. The cluster representative sequences without a hit in the SILVA database were compared against the non-redundant database from NCBI using BLASTx with a bit score cut off of 35. The top hit of each BLASTx-search was used for the functional assignment of the cluster representatives.

The cluster representative sequences without a hit in the SILVA database were further scanned with profile hidden Markov models of the ModEnzA EC groups [69] and the Pfam protein families [70]. The Pfam-hits were converted to EC numbers and along with the ModEnzA EC-hits mapped to the Kyoto Encyclopedia of Genes and Genomes (KEGG) reference pathways using the FROMP pathway mapping and visualization tool [96]. For ease of data analysis all cluster representative sequences, including clustering information from Cd-hit (cluster identification number, cluster size and all clustered nucleotide sequences), results from BLAST-, Pfam- and EC-searches as well as the taxonomic assignment from MEGAN were added to a MySQL database [96].

### Sequencing statistics

Combining all DNA sequences, 0.3% of them were of rRNA gene origin. For the RNA sequences, the percentage of rRNA sequences varied between 58 and 76% with the exception of the surface sample collected at 5 m, which was dominated by eukaryotic rRNA sequences (>80%). The high count of eukaryotic rRNA sequences in the surface indicates that a further depletion of eukaryotic rRNAs supplementing the depletion of prokaryotic rRNAs would have been worthwhile. Nevertheless, also the depletion of prokaryotic rRNA still resulted in relatively high counts of bacterial rRNAs (7–55%), whereas the number of archaeal rRNAs was mostly negligible (2–11%). The rRNAs and rRNA genes were excluded from further analysis (except for Figure 3A), leaving 1,882,842 DNA and 421,528 RNA sequences. From this pool, 54% (DNA) and 53% (RNA) of the sequences could be identified as protein-coding (matching either the non-redundant database of NCBI or profile hidden Markov model scans of the ModEnzA EC groups or the Pfam protein families); the remainder could not be assigned. Of all protein-coding sequences, 82% of the DNA and 99% of the RNA sequences could be taxonomically identified (matched the non-redundant database of NCBI).

### Hierarchical clustering of sequences using the taxonomic profiles

The taxonomic profiles of the metagenomes and the metatranscriptomes (the occurrence of the microbial taxa in the samples as a percentage of the total number of sequences having a BLASTx-hit) as presented in Figure 3 were used for a hierarchical clustering with the PRIMER 6 program [102]. The samples were grouped into 6 categories according to the depths and a multivariate statistical test (ANOSIM) was used to determine if the groupings were distinct from each other in terms of their microbial communities. The relationships between the depth groups were visualized in a non-parametric Multidimensional Scaling plot using PRIMER 6.

### Metabolic and taxonomic diversity measures

The EC activity matrix (with sample sizes equalized to the smallest sample) was exported from the FROMP pathway mapping tool [96] and the EC counts for each sample were used to calculate the inverse of Simpson's index (1/D) where D = Σ P_i_^2^ and P_i_ representing the proportional abundance of species i, and the Evenness E = (1/D)/S with S being the number of unique species. Similar calculations were also performed for the taxonomic assignments (as presented in Figure 3) from the BLASTx-searches normalized to total number of sequences having a BLASTx-hit.

### Sequence recruitment onto reference genomes

The sequence data was recruited onto the reference genomes of the five most abundant organisms (as detected with BLAST-searches) SUP05, *R. magnifica*, *V. okutanii*, *Sulfurovum* and *D. autotrophicum* using the MUMmer program [103]. For SUP05, the draft genome (the metagenome with an ordered assembly of the contigs) as provided by Walsh and colleagues [41] was used and treated like the other genomes. The recruited sequences were re-assessed using a BLAST-search against the reference genomes. Sequences that hit more than one genome with a bit score difference of less than 5% between the first and second hits were discarded, giving rise to a non-overlapping set of sequences for each genome. These sequences were then recruited onto the genomes again to calculate the average coverage over non-overlapping windows of 300 base pairs. The coverage of RNA sequences in every reference genome window was normalized by the total number of BLASTx-hits for that metatranscriptome and divided by the coverage of DNA sequences (which have also been normalized by the total number of BLASTx-hits in that metagenome) for the same window. This value, the expression-ratio, was subsequently corrected for differences in sizes of the metatranscriptome and metagenome and selected regions of the reference genomes were plotted using customized R and PERL scripts.

### Flow-cytometry cell counts

Samples for flow-cytometry were fixed with a final concentration of 1% paraformaldehyde and stored at −80°C until analysis. Total microbial cell counts were performed on a FACSCalibur flow cytometer (BD Biosciences). After 20 minutes staining of the samples with SybrGreen (Qiagen) at 4°C, cells were counted for 2 minutes or until a total count of 50,000 was reached. Sample flow rate was calibrated with standard beads (Trucount, BD Biosciences) and cell numbers were calculated via the time of measurement.

### Chemical analysis and microsensor measurements

Our pump-CTD was equipped with a custom-built amperometric O_2_ microsensor to obtain vertical profiles of dissolved O_2_ (detection limit 0.5–1 µM). In addition, the recently developed self-calibrating Switchable Trace amount Oxygen (STOX) sensor was deployed, which allows high-accuracy O_2_ measurements in near anoxic environments (detection limit ∼50 nM during our measurements) [13], [16]. After a minimum of ten minute sensor equilibration at a given sampling depth, at least five measuring cycles were used to calculate O_2_ concentrations.

Water samples for nutrient analysis were taken with a depth resolution of 1 to 2 m. NH_4_^+^ was measured fluorometrically [104] and NO_2_^−^ was analyzed spectrophotometrically [105] on board. Water samples for NO_3_^−^ and PO_4_^3−^ were stored frozen until spectrophotometric determination [105] with an autoanalyzer (TRAACS 800, Bran & Lubbe). Detection limits for NH_4_^+^, NO_2_^−^, NO_3_^−^ and PO_4_^3−^ were 10, 10, 100 and 100 nmol l^−1^, respectively. Dissolved N_2_O concentrations were determined on board in triplicates measurements using the GC headspace equilibration method as described elsewhere [106].

H_2_S concentrations were measured continuously on water sampled from the pump-CTD using a custom-built microsensor with a detection limit of ∼0.5 µM [107]. Chemically determined H_2_S concentration (both H_2_S and HS^−^; detection limit ∼0.5 µM) on discrete water samples were used to calibrate the microsensor [108]. Although the sulfidic waters contained a composite of H_2_S and HS^−^, we use the term H_2_S throughout the manuscript to avoid unnecessary complexity.

Using a 125 m depth cut off and a grid resolution of 1′ (bathymetrical data was obtained from the National Geophysical Data Center) along the ∼200 km cruise track on the Peruvian shelf where H_2_S was detected in the water column, a total area of ∼5500 km^2^ was calculated to be affected by the sulfidic plume. The shelf contained mean H_2_S-maxima close to the bottom of the water column of 3.4 µM. H_2_S was extending vertically over the water column with an averaged depth of ∼80 m, yielding ∼440 km^3^ of H_2_S-containing waters. Based on an average H_2_S concentration of 1.5 µM, we estimated a total content of ∼2.2×10^4^ tons H_2_S within the plume.

### Flux calculations

The density was calculated using the data processing program SeaSoft (Sea-Bird Electronics). The stability of the water column was expressed using the Brunt-Väisälä frequency N, defined as: N^2^ = −(g/ρ)×(∂ρ/∂z) where g is the gravitational acceleration, ρ the water density and z the water depth. The density gradient was calculated over 4 m bins. The turbulent diffusivity Ez was calculated as described earlier [109] from the Brunt-Väisälä frequency and the dissipation rate of turbulent kinetic energy ε: Ez = γε/N^2^ with the mixing coefficient γ = 0.2. We applied a mean ε of 1.85×10^−9^ W kg^−1^ [110]. This value was measured for the open-ocean thermocline [110] and was applied in several rate diffusion models [111], [112]. Vertical concentration gradients for O_2_, H_2_S, and NO_3_^−^ were calculated over 4 m bins. Fluxes of O_2_, H_2_S, and NO_3_^−^ at respective depths were calculated according to Fick's law: J_i_ = Ez×(∂C/∂z).

### Satellite images

Data of the sensors MODIS (Moderate Resolution Imaging Spectroradiometer) aboard the satellites Aqua and Terra (NASA) as well as MERIS (Medium Resolution Imaging Spectrometer) aboard the satellite Envisat (ESA) were implemented to study milky turquoise discolouration in waters off the Peruvian coast. Cloudy weather north of Pisco during most of our cruise made remote sensing difficult, but turquoise discolorations were observed off Lima on January 20–21^st^ (Figure S1A) and 27–28^th^ (Figure S1B). The estimation of the extent of the plumes requires data of higher spatial resolution; the algorithm for the identification of colloidal S^0^ plumes is based on the high spectral resolution of MERIS data [57]. Since full resolution MERIS data is not available for this region we present a high resolution MODIS true colour image in our main figure (Figure 1E).

However, the reflectance spectra derived from reduced resolution MERIS data (Figure S1C), revealed that the turquoise plume southwest of Pisco conformed the criteria for S^0^ in the identification algorithm distinguishable from optically similar coccolithophore blooms [113]. Differences in the shape and appearance of the colloidal S^0^ plume on the MERIS image (Figure S1C) and the MODIS images (Figure 1E) from May 7^th^ and 8^th^ demonstrate the temporal variability, which is also visible in changes in the cloud structure between the two images.

### Rate measurements of nitrogen transformation processes

Rates of microbial nitrogen transformations were measured at three or four depths in ^15^N-labeling experiments as described previously [114], [115]. Briefly, nitrogen-loss via anammox and denitrification as well as dissimilatory NO_3_^−^ reduction to NO_2_^−^ and NH_4_^+^ were measured in short-term incubation experiments amended with either ^15^NO_3_, ^15^NO_2_^−^, ^15^N_2_O or ^15^NH_4_^+^ (20, 10, 1 and 5 µmol l^−1^, respectively; isotopes: Campro Scientific). Time-series incubations were carried out in 12 ml Exetainers (Labco) and biological activity was stopped in one replicate Exetainer at each time interval (0, 6, 12, 24 and 48 h) by the addition of saturated mercuric chloride. Anammox and denitrification was measured as the production of ^15^N-labeled N_2_ in ^15^NO_2_^−^ and ^15^NO_3_^−^, ^15^NO_2_^−^ and ^15^N_2_O incubations, respectively. The N-isotopic composition of N_2_ gas produced in these experiments was determined by gas chromatography isotope-ratio mass spectrometry (GC/IRMS, Fisons VG Optima) [115]. NO_2_^−^ produced from ^15^NO_3_^−^ and NH_4_^+^ produced from ^15^NO_2_^−^ was determined by GC/IRMS after conversion of NO_2_^−^ and NH_4_^+^ to N_2_ by sulfamic acid [114] and alkaline hypobromite [24], respectively. Production rates were calculated from the increase of ^15^N-concentrations over time and only significant and linear productions of ^15^N-species without a lag-phase were considered (*t*-tests, *p*<0.05; R^2^>0.8). Rates are presented as net production rates corrected for the mole fractions of ^14^N in the original substrate pools.

### Rate measurements of carbon fixation

 Triplicate incubations of 4.5 l seawater were set up with water from 5, 15, 30, 80 and 100 m. To each bottle 4.5 ml of ^13^C bicarbonate solution (1 g ^13^C bicarbonate in 50 ml water) was added and bottles were incubated in on-deck incubators shaded to 25% surface irradiance with blue lagoon light foil (Lee Filters) and continuously cooled with surface seawater (5 and 15 m) or incubated at 12°C in the dark (30, 80 and 100 m) for 24 hours. At the end of the incubation 1–2 l were filtered on precombusted (450°C for 6 hours) Whatman GF/F filters (as these filters have an average pore size of 0.7 µm and small microorganisms may have passed through, the calculated CO_2_ fixation rates we present here have to be considered as minimal rates). The filters were oven dried (50°C for 24 hours) and stored for later analysis at room temperature. Filters were smoked overnight with 37% HCl to remove inorganic carbon retained on the filters, dried for 2 hours at 50°C and then analyzed in a CHN analyzer coupled to an isotope ratio monitoring mass spectrometer. The CO_2_ fixation rate was calculated according to the enrichment of ^13^C in the samples relative to unlabeled background values: C_fix_ = (At%_sample_−tAt%_background_)/(At%_label_−At%_background_)×(POC/time) where At%_sample_ is the ratio of ^13^C/^12^C times 100 in the particulate organic carbon pool (POC), At%_background_ is the same ratio in unlabeled POC and At%_label_ is the final ratio of ^13^C/^12^C in the incubation bottle after label addition. The resulting ratio is multiplied with the concentration of POC and divided by the incubation time in days. Since we did not perform killed controls, we cannot exclude or estimate the contribution of anapleurotic carbon fixation in our samples. We averaged the CO_2_ fixation over the predicted predominantly photic (0–20 m, ∼14.4 µmol C l^−1^ d^−1^) and aphotic (20–100 m, ∼1.2 µmol C l^−1^ d^−1^, dark incubations) zones by multiplying the rates with the respective water depths. A photic zone CO_2_ fixation of ∼288 mmol C m^−2^ d^−1^ and a light-independent CO_2_ fixation of ∼96 mmol C m^−2^ d^−1^ was estimated.

When compared to the overall mean CO_2_ fixation rate of the Humboldt Current System off Peru (2.18 g C m^−2^ d^−1^ or 182 mmol C m^−2^ d^−1^ [2] and 3.5 g C m^−2^ d^−1^ or 292 mmol C m^−2^ d^−1^ [4]), our measured dark CO_2_ fixation (over an 80 m deep aphotic zone) contributed 33–53% of the total CO_2_ fixation. Extrapolating the dark CO_2_ fixation rates over the entire sulfidic plume (∼5500 km^2^), we calculated a total CO_2_ fixation of ∼5.3×10^8^ mol C d^−1^ or ∼6.3×10^3^ tons C d^−1^. This CO_2_ fixation estimate would contribute 1.2% of the total primary production of the Humboldt Current System off the Peruvian coast as presented by Carr, 2002 (∼2×10^8^ tons C y^−1^ or ∼5.5×10^5^ tons C d^−1^). The average CO_2_ fixation rates per cell were calculated from total microbial cell counts as obtained from flow-cytometry.

### Accession numbers of sequence data

Metagenomic and metatranscriptomic sequences have been deposited in the metagenomics analysis server (MG-RAST) under accession numbers 4460677.3, 4450892.3, 4450891.3, 4460736.3, 4461588.3, 4460676.3, 4452038.3, 4460734.3, 4452039.3, 4452042.3, 4460735.3, 4460734.3 and 4452043.3.

## Supporting Information

Figure S1 **Satellite images of the Peruvian coast.** The red circles mark colloidal S^0^ plumes. (A) Satellite image (MODIS) of the area around Lima on January, 29^th^, 2009. (B) Satellite image (MODIS) of the area around Pisco on January, 27^th^, 2009. (C) Satellite image (MERIS) of the area around Pisco on May 7^th^, 2009.
(TIF)

Figure S2 **Multivariate statistical analysis and clustering of all protein-coding sequences based on shared taxonomic categories.** Taxonomic categories are chosen according to Figure 3. (A) Hierarchical clustering. (B) Non-parametric Multidimensional Scaling. Plot is labelled by prior groupings of the samples. The solid green circles mark the hierarchical clusters obtained using a similarity cut off of 86%. (C) ANOSIM test for significance of difference between the prior groupings.
(TIFF)

Figure S3 **Vertical distribution of the most abundant taxa.** Shown are the eight most abundant organisms (on the highest taxonomic level possible) in percent of all sequences in the DNA and RNA datasets (excluding rRNA genes and rRNAs); ordered descending according to DNA counts and supplemented with the remainder of the top eight organisms from the RNA dataset if not already present in the DNA dataset. Please note that no RNA sequences were identified as similar to the SUP05 cluster bacterium at 20 and 40 m with BLASTx-searches against the non-redundant database of NCBI.
(PDF)

Figure S4 **Vertical distribution of sequences recruited onto the genomes of (A)** ***Sulfurovum*** **sp. NBC37- 1 and (B)** ***Desulfobacterium autotrophicum*** **HRM2.** Shown are selected genes encoding for enzymes involved in oxygen- (blue), sulfur- (yellow), nitrogen- (red), carbon- (green) and hydrogen-metabolism (purple) in the corresponding order of the genomes. The y-axis depicts the log of the expression-ratio, a measure for the selective enrichment of transcripts over the corresponding gene, normalized to the total pool of protein-coding sequences. A list of the start and end position of each gene and the full name of the corresponding enzyme are shown in Table S3.
(TIFF)

Figure S5 **Vertical distribution of the most abundant functional assignments.** Shown are the top five most abundant EC numbers in percent of all protein-coding sequences in the DNA and RNA datasets; ordered descending according to the DNA counts and supplemented with the remainder of the top five EC numbers from the RNA dataset if not already present in the DNA dataset. Please note that the data presented here is based only on EC number- and Pfam-assignments; BLAST-hits are not included.
(PDF)

Table S1 **Sequencing statistics.** ^a^as obtained with Cd-hit. ^b^as obtained by BLASTn-searches against the SILVA database. ^c^as obtained by BLASTx-searches against the non-redundant database of NCBI and by scans with profile hidden Markov models of the ModEnzA EC groups and of the Pfam protein families. ^d^average.
(DOC)

Table S2 **Metabolic and taxonomic evenness and diversity in all protein-coding sequences.** A collection of all EC number- and Pfam-assignments was used to determine the metabolic diversity, while the taxonomic diversity was calculated using all hits from BLASTx-searches. Shown are the evenness and the diversity (inverse of the Simpson's index) for both the metagenomic and metatranscriptomic datasets.
(DOC)

Table S3 **Genomic regions of the sequences recruited onto proteobacterial genomes as plotted in** **Figures 5** **and S4.** Shown are the start and end position of each gene and the corresponding enzyme name for the uncultured SUP05 cluster bacterium, *Candidatus* Ruthia magnifica str. Cm, *Candidatus* Vesicomyosocius okutanii HA, *Sulfurovum* sp. NBC37- 1 and *Desulfobacterium autotrophicum* HRM2.
(DOC)

## References

[1] Friederich GE, Codispoti LA (1987) An analysis of continuous vertical nutrient profiles taken during a cold-anomaly off Peru. Deep-Sea Res 34 (5–6) 1049–1065.

[2] Carr ME (2002) Estimation of potential productivity in Eastern Boundary Currents using remote sensing. Deep-Sea Res Pt II 49 (1–3) 59–80.

[3] Chavez FP, Messie M (2009) A comparison of Eastern Boundary Upwelling Ecosystems. Prog Oceanogr 83 (1–4) 80–96.

[4] Montecino V, Lange CB (2009) The Humboldt Current System: Ecosystem components and processes, fisheries, and sediment studies. Prog Oceanogr 83 (1–4) 65–79.

[5] Ryther JH (1969) Photosynthesis and fish production in the sea. Science 166 (3901) 72–76. 5817762 10.1126/science.166.3901.72

[6] Pauly D, Christensen V (1995) Primary Production Required to Sustain Global Fisheries. Nature 374 (6519) 255–257.

[7] Wyrtki K (1962) The Oxygen Minima in Relation to Ocean Circulation. Deep-Sea Res 9 (1) 11–23.

[8] Wright JJ, Konwar KM, Hallam SJ (2012) Microbial ecology of expanding oxygen minimum zones. Nat Rev Microbiol 10 (6) 381–394. 22580367 10.1038/nrmicro2778

[9] Karstensen J, Stramma L, Visbeck M (2008) Oxygen minimum zones in the eastern tropical Atlantic and Pacific oceans. Prog Oceanogr 77 (4) 331–350.

[10] Helly JJ, Levin LA (2004) Global distribution of naturally occurring marine hypoxia on continental margins. Deep-Sea Res Pt I 51 (9) 1159–1168.

[11] Stramma L, Johnson GC, Sprintall J, Mohrholz V (2008) Expanding Oxygen-Minimum Zones in the Tropical Oceans. Science 320 (5876) 655–658. 18451300 10.1126/science.1153847

[12] Paulmier A, Ruiz-Pino D (2009) Oxygen minimum zones (OMZs) in the modern ocean. Prog Oceanogr 80 (3–4) 113–128.

[13] Revsbech NP, Larsen LH, Gundersen J, Dalsgaard T, Ulloa O, et al (2009) Determination of ultra-low oxygen concentrations in oxygen minimum zones by the STOX sensor. Limnol Oceanogr Meth 7: 371–381.

[14] Canfield DE, Stewart FJ, Thamdrup B, De Brabandere L, Dalsgaard T, et al (2010) A Cryptic Sulfur Cycle in Oxygen-Minimum-Zone Waters off the Chilean Coast. Science 330 (6009) 1375–1378. 21071631 10.1126/science.1196889

[15] Jensen MM, Lam P, Revsbech NP, Nagel B, Gaye B, et al (2011) Intensive nitrogen loss over the Omani Shelf due to anammox coupled with dissimilatory nitrite reduction to ammonium. ISME J 5 (10) 1660–1670. 21509044 10.1038/ismej.2011.44 PMC3176517

[16] Kalvelage T, Jensen MM, Contreras S, Revsbech NP, Lam P, et al (2011) Oxygen Sensitivity of Anammox and Coupled N-Cycle Processes in Oxygen Minimum Zones. PLoS One 7 (5) e37118 doi:37110.31371/journal.pone.0037118 10.1371/journal.pone.0029299 PMC3247244 22216239

[17] Emery KO, Orr WL, Rittenberg SC (1955) Nutrient budgets in the ocean. In: Essays in Natural Sciences in Honor of Captain Allan Handcock. Los Angeles: University of Southern California Press. pp. 299–309.

[18] Codispoti LA, Brandes JA, Christensen JP, Devol AH, Naqvi SWA, et al (2001) The oceanic fixed nitrogen and nitrous oxide budgets: Moving targets as we enter the anthropocene? Sci Mar 65: 85–105.

[19] Gruber N (2004) The dynamics of the marine nitrogen cycle and atmospheric CO_2_. In: Oguz T, Follows M, editors. Carbon Climate Interactions. NATO ASI Series. Dordrecht: Kluwer Academic. pp. 97–148.

[20] Dalsgaard T, Thamdrup B, Farias L, Revsbech NP (2012) Anammox and denitrification in the oxygen minimum zone of the eastern South Pacific. Limnol Oceanogr 57 (5) 1331–1346.

[21] Ward BB, Devol AH, Rich JJ, Chang BX, Bulow SE, et al (2009) Denitrification as the dominant nitrogen loss process in the Arabian Sea. Nature 461 (7260) 78–81. 19727197 10.1038/nature08276

[22] Kuypers MMM, Lavik G, Woebken D, Schmid M, Fuchs BM, et al (2005) Massive nitrogen loss from the Benguela upwelling system through anaerobic ammonium oxidation. Proc Natl Acad Sci U S A 102 (18) 6478–6483. 15843458 10.1073/pnas.0502088102 PMC556276

[23] Hamersley MR, Lavik G, Woebken D, Rattray JE, Lam P, et al (2007) Anaerobic ammonium oxidation in the Peruvian oxygen minimum zone. Limnol Oceanogr 52 (3) 923–933.

[24] Lam P, Lavik G, Jensen MM, van de Vossenberg J, Schmid M, et al (2009) Revising the nitrogen cycle in the Peruvian oxygen minimum zone. Proc Natl Acad Sci U S A 106 (12) 4752–4757. 19255441 10.1073/pnas.0812444106 PMC2649953

[25] Kalvelage T, Lavik G, Lam P, Contreras S, Arteaga L, et al (2013) Nitrogen cycling driven by organic matter export in the South Pacific oxygen minimum zone. Nature Geosci 6 (3) 228–234.

[26] Thamdrup B, Dalsgaard T, Jensen MM, Ulloa O, Farias L, et al (2006) Anaerobic ammonium oxidation in the oxygen-deficient waters off northern Chile. Limnol Oceanogr 51 (5) 2145–2156.

[27] Diaz RJ, Rosenberg R (2008) Spreading dead zones and consequences for marine ecosystems. Science 321 (5891) 926–929. 18703733 10.1126/science.1156401

[28] Ward BB, Glover HE, Lipschultz F (1989) Chemoautotrophic activity and nitrification in the oxygen minimum zone off Peru. Deep-Sea Res 36 (7) 1031–1051.

[29] Jørgensen BB (1982) Ecology of the bacteria of the sulphur cycle with special reference to anoxic-oxic interface environments. Philos Trans R Soc Lond B 298 (1093) 543–561. 6127739 10.1098/rstb.1982.0096

[30] Bruchert V, Jørgensen BB, Neumann K, Riechmann D, Schlosser M, et al (2003) Regulation of bacterial sulfate reduction and hydrogen sulfide fluxes in the central Namibian coastal upwelling zone. Geochim Cosmochim Ac 67 (23) 4505–4518.

[31] Lavik G, Stuhrmann T, Bruchert V, Van der Plas A, Mohrholz V, et al (2008) Detoxification of sulphidic African shelf waters by blooming chemolithotrophs. Nature 457 (7229) 581–584. 19078958 10.1038/nature07588

[32] Shao MF, Zhang T, Fang HHP (2010) Sulfur-driven autotrophic denitrification: diversity, biochemistry, and engineering applications. Appl Microbiol Biotechnol 88 (5) 1027–1042. 20809074 10.1007/s00253-010-2847-1

[33] Orcutt BN, Sylvan JB, Knab NJ, Edwards KJ (2011) Microbial Ecology of the Dark Ocean above, at, and below the Seafloor. Microbiol Mol Biol Rev 75 (2) 361–422. 21646433 10.1128/MMBR.00039-10 PMC3122624

[34] Dugdale RC, Goering JJ, Barber RT, Smith RL, Packard TT (1977) Denitrification and Hydrogen-Sulfide in Peru Upwelling Region during 1976. Deep-Sea Res 24 (6) 601–608.

[35] Naqvi SW, Jayakumar DA, Narvekar PV, Naik H, Sarma VV, et al (2000) Increased marine production of N_2_O due to intensifying anoxia on the Indian continental shelf. Nature 408 (6810) 346–349. 11099038 10.1038/35042551

[36] Hannig M, Lavik G, Kuypers MMM, Woebken D, Martens-Habbena W, et al (2007) Shift from denitrification to anammox after inflow events in the central Baltic Sea. Limnol Oceanogr 52 (4) 1336–1345.

[37] Stewart FJ, Ulloa O, DeLong EF (2011) Microbial metatranscriptomics in a permanent marine oxygen minimum zone. Environ Microbiol 14 (1) 23–40. 21210935 10.1111/j.1462-2920.2010.02400.x

[38] Stevens H, Ulloa O (2008) Bacterial diversity in the oxygen minimum zone of the eastern tropical South Pacific. Environ Microbiol 10 (5) 1244–1259. 18294206 10.1111/j.1462-2920.2007.01539.x

[39] Ulloa O, Canfield DE, DeLong EF, Letelier RM, Stewart FJ (2012) Microbial oceanography of anoxic oxygen minimum zones. Proc Natl Acad Sci U S A 109 (40) 15996–16003. 22967509 10.1073/pnas.1205009109 PMC3479542

[40] Stewart FJ, Dalsgaard T, Young CR, Thamdrup B, Revsbech NP, et al (2012) Experimental Incubations Elicit Profound Changes in Community Transcription in OMZ Bacterioplankton. PLoS One 7 (5) e37118 doi:37110.31371/journal.pone.0037118 22615914 10.1371/journal.pone.0037118 PMC3353902

[41] Walsh DA, Zaikova E, Howes CG, Song YC, Wright JJ, et al (2009) Metagenome of a Versatile Chemolithoautotroph from Expanding Oceanic Dead Zones. Science 326 (5952) 578–582. 19900896 10.1126/science.1175309

[42] Glaubitz S, Labrenz M, Jost G, Jürgens K (2010) Diversity of active chemolithoautotrophic prokaryotes in the sulfidic zone of a Black Sea pelagic redoxcline as determined by rRNA-based stable isotope probing. FEMS Microbiol Ecol 74 (1) 32–41. 20649907 10.1111/j.1574-6941.2010.00944.x

[43] Glaubitz S, Lueders T, Abraham WR, Jost G, Jürgens K, et al (2009) ^13^C-isotope analyses reveal that chemolithoautotrophic *Gamma-* and *Epsilonproteobacteria* feed a microbial food web in a pelagic redoxcline of the central Baltic Sea. Environ Microbiol 11 (2) 326–337. 18793316 10.1111/j.1462-2920.2008.01770.x

[44] Brettar I, Rheinheimer G (1991) Denitrification in the Central Baltic: evidence for H_2_S-oxidation as motor of denitrification at the oxic-anoxic interface. Mar Ecol-Prog Ser 77 (2–3) 157–169.

[45] Brettar I, Labrenz M, Flavier S, Botel J, Kuosa H, et al (2006) Identification of a *Thiomicrospira denitrificans*-Like Epsilonproteobacterium as a Catalyst for Autotrophic Denitrification in the Central Baltic Sea. Appl Environ Microbiol 72 (2) 1364–1372. 16461688 10.1128/AEM.72.2.1364-1372.2006 PMC1392969

[46] Jørgensen BB, Fossing H, Wirsen CO, Jannasch HW (1991) Sulfide oxidation in the anoxic Black Sea chemocline. Deep-Sea Res 38: S1083–S1103.

[47] Luther GW, Church TM, Powell D (1991) Sulfur speciation and sulfide oxidation in the water column of the Black Sea. Deep-Sea Res 38: S1121–S1137.

[48] Sorokin YI, Sorokin PY, Avdeev VA, Sorokin DY, Ilchenko SV (1995) Biomass, Production and Activity of Bacteria in the Black-Sea, with Special Reference to Chemosynthesis and the Sulfur Cycle. Hydrobiologia 308 (1) 61–76.

[49] Zhang JZ, Millero FJ (1993) The Chemistry of the Anoxic Waters in the Cariaco Trench. Deep-Sea Res Pt I 40 (5) 1023–1041.

[50] Hayes MK, Taylor GT, Astor Y, Scranton MI (2006) Vertical distributions of thiosulfate and sulfite in the Cariaco Basin. Limnol Oceanogr 51 (1) 280–287.

[51] Tebo BM, Emerson S (1986) Microbial manganese(II) oxidation in the marine environment: a quantitative study. Biogeochemistry 2 (2) 149–161.

[52] Burtt J (1852) On fish destroyed by sulphuretted hydrogen in the Bay of Callao. AM J SCI 2 (13) 433–434.

[53] Hamukuaya H, O'Toole MJ, Woodhead PMJ (1998) Observations of severe hypoxia and offshore displacement of Cape hake over the Namibian shelf in 1994. S Afr J Mar Sci 19: 57–59.

[54] Hart TJ, Currie RI (1960) The Benguela Current. In: Discovery Report 31. Cambridge: Cambridge University Press. pp. 123–298.

[55] Copenhagen WJ (1953) The periodic mortality of fish in the Walvis region - A phenomenon within the Benguela Current. S Afr Div Sea Fish Invest Rep 14: 1–35.

[56] Weeks SJ, Currie B, Bakun A (2002) Massive emissions of toxic gas in the Atlantic. Nature 415 (6871) 493–494. 10.1038/415493b 11823847

[57] Ohde T, Siegel H, Reissmann J, Gerth M (2007) Identification and investigation of sulphur plumes along the Namibian coast using the MERIS sensor. Cont Shelf Res 27 (6) 744–756.

[58] Frias-Lopez J, Shi Y, Tyson GW, Coleman ML, Schuster SC, et al (2008) Microbial community gene expression in ocean surface waters. Proc Natl Acad Sci U S A 105 (10) 3805–3810. 18316740 10.1073/pnas.0708897105 PMC2268829

[59] Shi Y, Tyson GW, DeLong EF (2009) Metatranscriptomics reveals unique microbial small RNAs in the ocean's water column. Nature 459 (7244) 266–269. 19444216 10.1038/nature08055

[60] Gifford SM, Sharma S, Rinta-Kanto JM, Moran MA (2010) Quantitative analysis of a deeply sequenced marine microbial metatranscriptome. ISME J 5 (3) 461–472. 20844569 10.1038/ismej.2010.141 PMC3105723

[61] Fuchs BM, Woebken D, Zubkov MV, Burkill P, Amann R (2005) Molecular identification of picoplankton populations in contrasting waters of the Arabian Sea. Aquat Microb Ecol 39 (2) 145–157.

[62] Lavin P, Gonzalez B, Santibanez JF, Scanlan DJ, Ulloa O (2010) Novel lineages of Prochlorococcus thrive within the oxygen minimum zone of the eastern tropical South Pacific. Environ Microbiol Rep 2 (6) 728–738. 23766277 10.1111/j.1758-2229.2010.00167.x

[63] Sunamura M, Higashi Y, Miyako C, Ishibashi J, Maruyama A (2004) Two bacteria phylotypes are predominant in the Suiyo seamount hydrothermal plume. Appl Environ Microbiol 70 (2) 1190–1198. 14766605 10.1128/AEM.70.2.1190-1198.2004 PMC348851

[64] Kuwahara H, Yoshida T, Takaki Y, Shimamura S, Nishi S, et al (2007) Reduced genome of the thioautotrophic intracellular symbiont in a deep-sea clam, *Calyptogena okutanii* . Curr Biol 17 (10) 881–886. 17493812 10.1016/j.cub.2007.04.039

[65] Newton ILG, Woyke T, Auchtung TA, Dilly GF, Dutton RJ, et al (2007) The Calyptogena magnifica chemoautotrophic symbiont genome. Science 315 (5814) 998–1000. 17303757 10.1126/science.1138438

[66] Nakagawa S, Takaki Y, Shimamura S, Reysenbach AL, Takai K, et al (2007) Deep-sea vent epsilon-proteobacterial genomes provide insights into emergence of pathogens. Proc Natl Acad Sci U S A 104 (29) 12146–12150. 17615243 10.1073/pnas.0700687104 PMC1907315

[67] Strittmatter AW, Liesegang H, Rabus R, Decker I, Amann J, et al (2009) Genome sequence of *Desulfobacterium autotrophicum* HRM2, a marine sulfate reducer oxidizing organic carbon completely to carbon dioxide. Environ Microbiol 11 (5) 1038–1055. 19187283 10.1111/j.1462-2920.2008.01825.x PMC2702500

[68] Brysch K, Schneider C, Fuchs G, Widdel F (1987) Lithoautotrophic growth of sulfate-reducing bacteria, and description of *Desulfobacterium autotrophicum* gen. nov., sp. nov. Arch Microbiol 148 (4) 264–274.

[69] Desai DK, Nandi S, Srivastava PK, Lynn AM (2011) ModEnzA: Accurate Identification of Metabolic Enzymes Using Function Specific Profile HMMs with Optimised Discrimination Threshold and Modified Emission Probabilities. Adv Bioinformatics doi:10.1155/2011/743782 10.1155/2011/743782 PMC3085309 21541071

[70] Finn RD, Mistry J, Tate J, Coggill P, Heger A, et al (2010) The Pfam protein families database. Nucleic Acids Res 38: D211–222 doi:210.1093/nar/gkp1985 19920124 10.1093/nar/gkp985 PMC2808889

[71] Albert DB, Taylor C, Martens CS (1995) Sulfate reduction rates and low molecular weight fatty acid concentrations in the water column and surficial sediments of the Black Sea. Deep-Sea Res Pt I 42 (7) 1239–1260.

[72] Meyer B, Kuever J (2007) Molecular analysis of the distribution and phylogeny of dissimilatory adenosine-5′-phosphosulfate reductase-encoding genes (*aprBA*) among sulfuroxidizing prokaryotes. Microbiology+ 153: 3478–3498. 17906146 10.1099/mic.0.2007/008250-0

[73] Meyer B, Kuever J (2007) Phylogeny of the alpha and beta subunits of the dissimilatory adenosine-5′-phosphosulfate (APS) reductase from sulfate-reducing prokaryotes - origin and evolution of the dissimilatory sulfate-reduction pathway. Microbiology+ 153: 2026–2044. 17600048 10.1099/mic.0.2006/003152-0

[74] Finster K, Liesack W, Thamdrup B (1998) Elemental Sulfur and Thiosulfate Disproportionation by *Desulfocapsa sulfoexigens* sp. nov., a New Anaerobic Bacterium Isolated from Marine Surface Sediment. Appl Environ Microbiol 64 (1) 119–125. 9435068 10.1128/aem.64.1.119-125.1998 PMC124681

[75] Fossing H (1990) Sulfate Reduction in Shelf Sediments in the Upwelling Region Off Central Peru. Cont Shelf Res 10 (4) 355–367.

[76] Niggemann J (2005) Composition and degradation of organic matter in sediments from the Peru-Chile upwelling region. Bremen: University of Bremen Press. 200 p.

[77] Nakagawa S, Takai K (2008) Deep-sea vent chemoautotrophs: diversity, biochemistry and ecological significance. FEMS Microbiol Ecol 65 (1) 1–14. 18503548 10.1111/j.1574-6941.2008.00502.x

[78] Yamamoto M, Nakagawa S, Shimamura S, Takai K, Horikoshi K (2010) Molecular characterization of inorganic sulfur-compound metabolism in the deep-sea epsilonproteobacterium *Sulfurovum* sp. NBC37-1. Environ Microbiol 12 (5) 1144–1152. 20132283 10.1111/j.1462-2920.2010.02155.x

[79] Pitcher RS, Watmough NJ (2004) The bacterial cytochrome *cbb* _3_ oxidases. Biochim Biophys Acta 1655 (1–3) 388–399. 15100055 10.1016/j.bbabio.2003.09.017

[80] Preisig O, Zufferey R, Thony-Meyer L, Appleby CA, Hennecke H (1996) A High-Affinity *cbb* _3_-Type Cytochrome Oxidase Terminates the Symbiosis-Specific Respiratory Chain of *Bradyrhizobium japonicum* . J Bacteriol 178 (6) 1532–1538. 8626278 10.1128/jb.178.6.1532-1538.1996 PMC177835

[81] Stolper DA, Revsbech NP, Canfield DE (2010) Aerobic growth at nanomolar oxygen concentrations. Proc Natl Acad Sci U S A 107 (44) 18755–18760. 20974919 10.1073/pnas.1013435107 PMC2973883

[82] Forte E, Urbani A, Saraste M, Sarti P, Brunori M, et al (2001) The cytochrome *cbb* _3_ from *Pseudomonas stutzeri* displays nitric oxide reductase activity. Eur J Biochem 268 (24) 6486–6491. 11737203 10.1046/j.0014-2956.2001.02597.x

[83] van der Oost J, Deboer APN, Degier JWL, Zumft WG, Stouthamer AH, et al (1994) The heme-copper oxidase family consists of three distinct types of terminal oxidases and is related to nitric oxide reductase. FEMS Microbiol Lett 121 (1) 1–9. 8082820 10.1111/j.1574-6968.1994.tb07067.x

[84] Grote J, Schott T, Bruckner CG, Glöckner FO, Jost G, et al (2012) Genome and physiology of a model Epsilonproteobacterium responsible for sulfide detoxification in marine oxygen depletion zones. Proc Natl Acad Sci U S A 109 (2) 506–510. 22203982 10.1073/pnas.1111262109 PMC3258601

[85] Wirsen CO, Sievert SM, Cavanaugh CM, Molyneaux SJ, Ahmad A, et al (2002) Characterization of an Autotrophic Sulfide-Oxidizing Marine *Arcobacter* sp. That Produces Filamentous Sulfur. Appl Environ Microbiol 68 (1) 316–325. 11772641 10.1128/AEM.68.1.316-325.2002 PMC126556

[86] Jensen MM, Kuypers MMM, Lavik G, Thamdrup B (2008) Rates and regulation of anaerobic ammonium oxidation and denitrification in the Black Sea. Limnol Oceanogr 53 (1) 23–36.

[87] Zamora LM, Oschlies A, Bange HW, Huebert KB, Craig JD, et al (2012) Nitrous oxide dynamics in low oxygen regions of the Pacific: insights from the MEMENTO database. Biogeosciences 9 (12) 5007–5022.

[88] Farias L, Fernandez C, Faundez J, Cornejo M, Alcaman ME (2009) Chemolithoautotrophic production mediating the cycling of the greenhouse gases N_2_O and CH_4_ in an upwelling ecosystem. Biogeosciences 6 (12) 3053–3069.

[89] Stewart FJ (2011) Dissimilatory sulfur cycling in oxygen minimum zones: an emerging metagenomics perspective. Biochem Soc Trans 39 (6) 1859–1863. 22103540 10.1042/BST20110708

[90] Grote J, Jost G, Labrenz M, Herndl GJ, Juergens K (2008) Epsilonproteobacteria Represent the Major Portion of Chemoautotrophic Bacteria in Sulfidic Waters of Pelagic Redoxclines of the Baltic and Black Seas. Appl Environ Microbiol 74 (24) 7546–7551. 18952879 10.1128/AEM.01186-08 PMC2607176

[91] Grote J, Labrenz M, Pfeiffer B, Jost G, Jürgens K (2007) Quantitative Distributions of *Epsilonproteobacteria* and a *Sulfurimonas* Subgroup in Pelagic Redoxclines of the Central Baltic Sea. Appl Environ Microbiol 73 (22) 7155–7161. 17921285 10.1128/AEM.00466-07 PMC2168200

[92] Jost G, Zubkov MV, Yakushev E, Labrenz M, Jürgens K (2008) High abundance and dark CO_2_ fixation of chemolithoautotrophic prokaryotes in anoxic waters of the Baltic Sea. Limnol Oceanogr 53 (1) 14–22.

[93] Jost G, Martens-Habbena W, Pollehne F, Schnetger B, Labrenz M (2009) Anaerobic sulfur oxidation in the absence of nitrate dominates microbial chemoautotrophy beneath the pelagic chemocline of the eastern Gotland Basin, Baltic Sea. FEMS Microbiol Ecol 71 (2) 226–236. 19925634 10.1111/j.1574-6941.2009.00798.x

[94] Detmer AE, Giesenhagen HC, Trenkel VM, Auf dem Venne H, Jochem FJ (1993) Phototrophic and heterotrophic pico- and nanoplankton in anoxic depths of the central Baltic Sea. Mar Ecol-Prog Ser 99 (1–2) 197–203.

[95] Chavez FP, Bertrand A, Guevara-Carrasco R, Soler P, Csirke J (2008) The northern Humboldt Current System: Brief history, present status and a view towards the future. Prog Oceanogr 79 (2–4) 95–105.

[96] Desai DK, Schunck H, Löser JW, LaRoche J (2013) Fragment recruitment on metabolic pathways: comparative metabolic profiling of metagenomes and metatranscriptomes. Bioinformatics 29 (6) 790–791. 23303511 10.1093/bioinformatics/bts721

[97] Li W, Godzik A (2006) Cd-hit: a fast program for clustering and comparing large sets of protein or nucleotide sequences. Bioinformatics 22 (13) 1658–1659. 16731699 10.1093/bioinformatics/btl158

[98] Altschul SF, Gish W, Miller W, Myers EW, Lipman DJ (1990) Basic Local Alignment Search Tool. J Mol Biol 215 (3) 403–410. 2231712 10.1016/S0022-2836(05)80360-2

[99] Pruesse E, Quast C, Knittel K, Fuchs BM, Ludwig W, et al (2007) SILVA: a comprehensive online resource for quality checked and aligned ribosomal RNA sequence data compatible with ARB. Nucleic Acids Res 35 (21) 7188–7196. 17947321 10.1093/nar/gkm864 PMC2175337

[100] Urich T, Lanzen A, Qi J, Huson DH, Schleper C, et al (2008) Simultaneous assessment of soil microbial community structure and function through analysis of the meta-transcriptome. PLoS One 3 (6) e2527 doi:2510.1371/journal.pone.0002527 18575584 10.1371/journal.pone.0002527 PMC2424134

[101] Huson DH, Auch AF, Qi J, Schuster SC (2007) MEGAN analysis of metagenomic data. Genome Res 17 (3) 377–386. 17255551 10.1101/gr.5969107 PMC1800929

[102] Clarke KR (1993) Nonparametric Multivariate Analyses of Changes in Community Structure. Aust J Ecol 18 (1) 117–143.

[103] Kurtz S, Phillippy A, Delcher AL, Smoot M, Shumway M, et al (2004) Versatile and open software for comparing large genomes. Genome Biol 5 (2) R12. 14759262 10.1186/gb-2004-5-2-r12 PMC395750

[104] Holmes RM, Aminot A, Kerouel R, Hooker BA, Peterson BJ (1999) A simple and precise method for measuring ammonium in marine and freshwater ecosystems. Can J Fish Aquat Sci 56 (10) 1801–1808.

[105] Grasshoff K, Kremling K, Ehrhardt M (1999) Methods of seawater analysis. Weinheim: Wiley-VCH. 600 p.

[106] Walter S, Bange HW, Breitenbach U, Wallace DWR (2006) Nitrous oxide in the North Atlantic Ocean. Biogeosciences 3 (4) 607–619.

[107] Jeroschewski P, Steuckart C, Kuhl M (1996) An amperometric microsensor for the determination of H_2_S in aquatic environments. Anal Chem 68 (24) 4351–4357.

[108] Cline JD (1969) Spectrophotometric Determination of Hydrogen Sulfide in Natural Waters. Limnol Oceanogr 14 (3) 454–458.

[109] Osborn TR (1980) Estimates of the Local Rate of Vertical Diffusion from Dissipation Measurements. J Phys Oceanogr 10 (1) 83–89.

[110] Gregg MC, Dasaro EA, Shay TJ, Larson N (1986) Observations of Persistent Mixing and Near-Inertial Internal Waves. J Phys Oceanogr 16 (5) 856–885.

[111] Lam P, Jensen MM, Lavik G, McGinnis DF, Muller B, et al (2007) Linking crenarchaeal and bacterial nitrification to anammox in the Black Sea. Proc Natl Acad Sci U S A 104 (17) 7104–7109. 17420469 10.1073/pnas.0611081104 PMC1849958

[112] Fennel W (1995) A Model of the Yearly Cycle of Nutrients and Plankton in the Baltic Sea. J Marine Syst 6 (4) 313–329.

[113] Siegel H, Ohde T, Gerth M, Lavik G, Leipe T (2007) Identification of coccolithophore blooms in the SE Atlantic Ocean off Namibia by satellites and in-situ methods. Cont Shelf Res 27 (2) 258–274.

[114] Füssel J, Lam P, Lavik G, Jensen MM, Holtappels M, et al (2011) Nitrite oxidation in the Namibian oxygen minimum zone. ISME J 6 (6) 1200–1209. 22170426 10.1038/ismej.2011.178 PMC3358024

[115] Holtappels M, Lavik G, Jensen MM, Kuypers MMM (2011) ^15^N-Labeling Experiments to Dissect the Contributions of Heterotrophic Denitrification and Anammox to Nitrogen Removal in the OMZ Waters of the Ocean. Method Enzymol 486: 223–251. 10.1016/B978-0-12-381294-0.00010-9 21185438

