# Supplementary figures and images for: Giant Hydrogen Sulfide Plume in the Oxygen Minimum Zone off Peru Supports Chemolithoautotrophy
Source: PLoS One. 2013 Aug 21;8(8):e68661. doi: 10.1371/journal.pone.0068661 (PMC3749208; doi:10.1371/journal.pone.0068661)

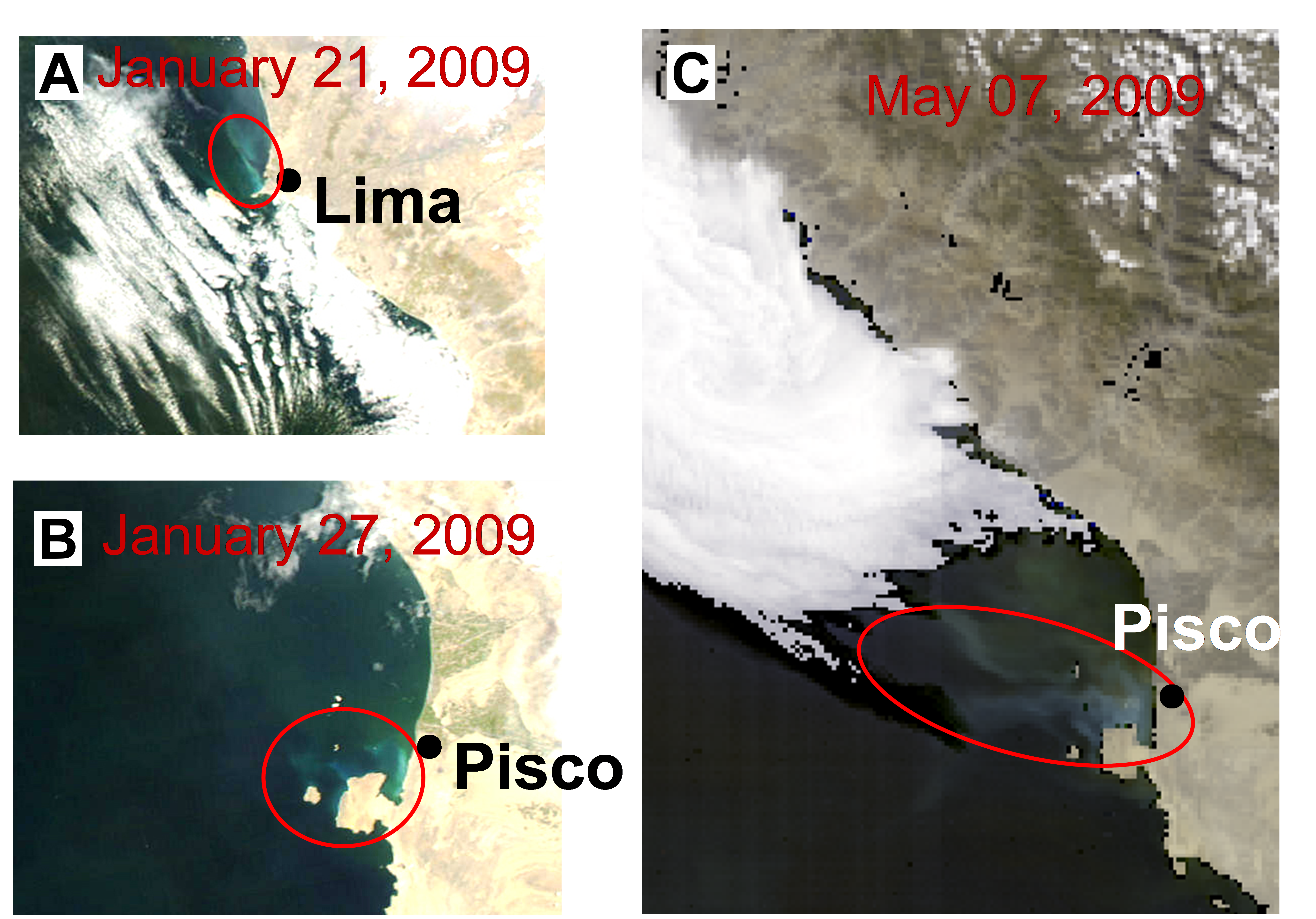

Supplement: Figure S1 — Satellite images of the Peruvian coast. The red circles mark colloidal S0 plumes. (A) Satellite image (MODIS) of the area around Lima on January, 29th, 2009. (B) Satellite image (MODIS) of the area around Pisco on January, 27th, 2009. (C) Satellite image (MERIS) of the area around Pisco on May 7th, 2009. (TIF) [file pone.0068661.s001.tif]

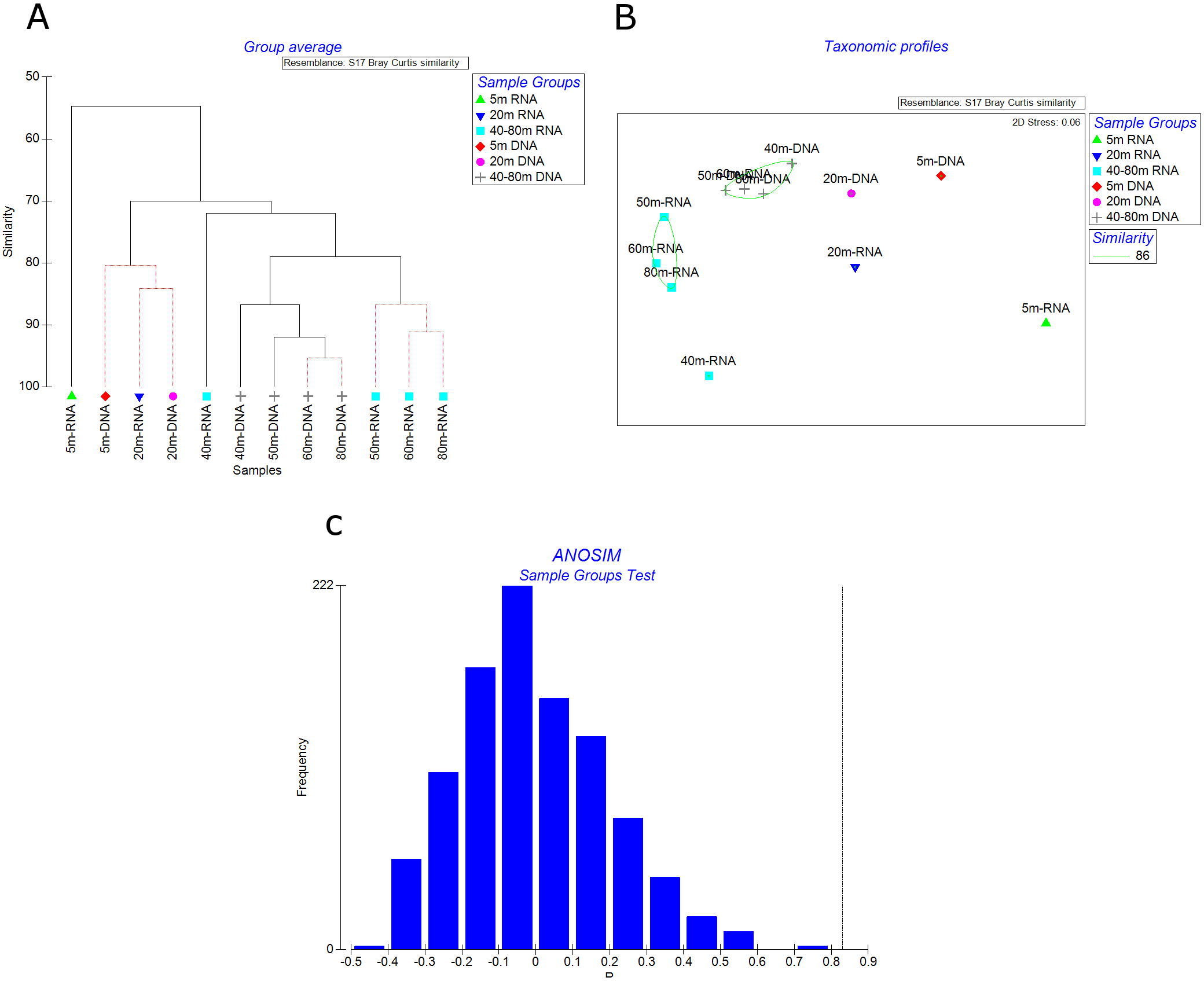

Supplement: Figure S2 — Multivariate statistical analysis and clustering of all protein-coding sequences based on shared taxonomic categories. Taxonomic categories are chosen according to Figure 3. (A) Hierarchical clustering. (B) Non-parametric Multidimensional Scaling. Plot is labelled by prior groupings of the samples. The solid green circles mark the hierarchical clusters obtained using a similarity cut off of 86%. (C) ANOSIM test for significance of difference between the prior groupings. (TIFF) [file pone.0068661.s002.tif]

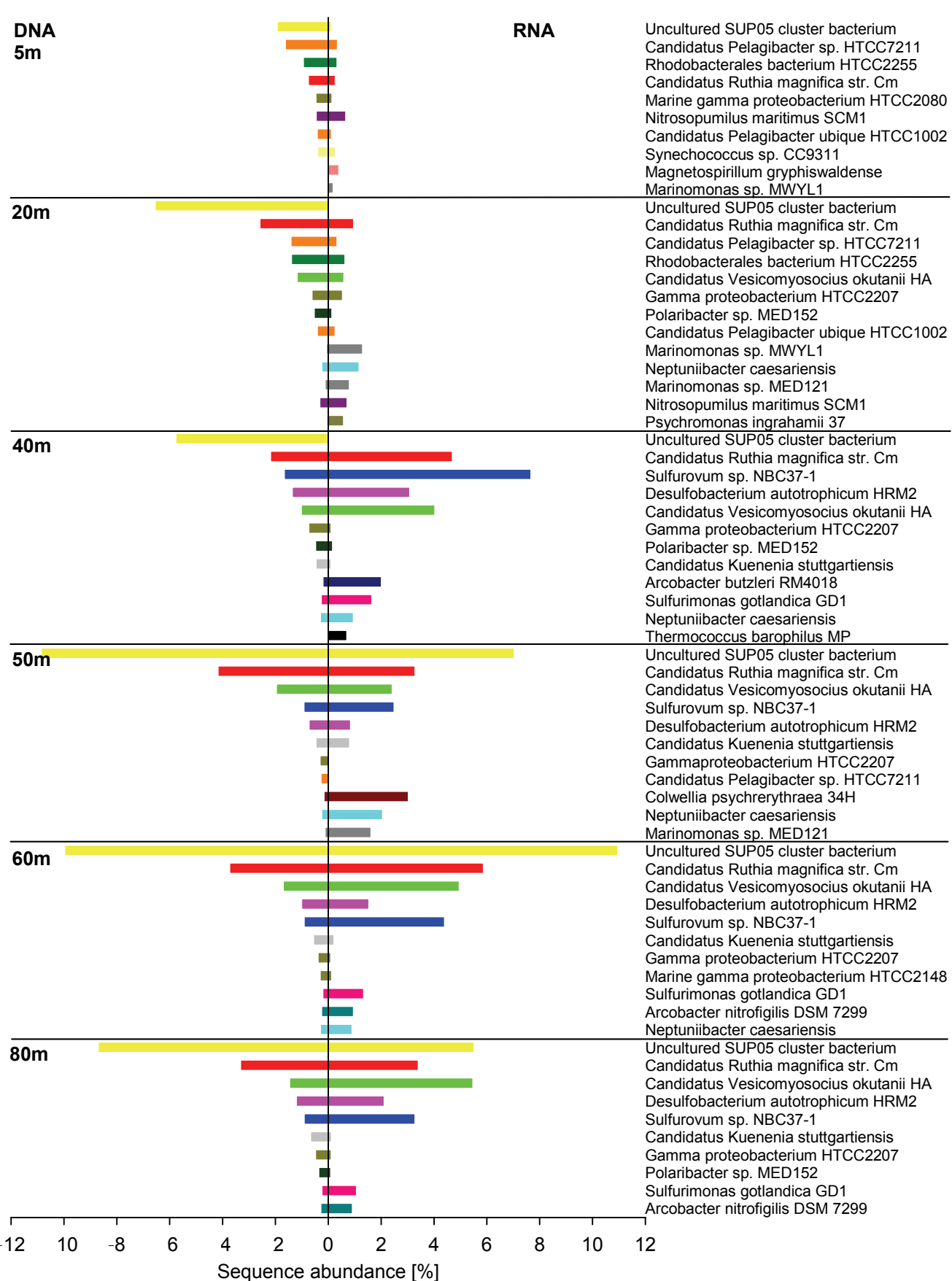

Supplement: Figure S3 — Vertical distribution of the most abundant taxa. Shown are the eight most abundant organisms (on the highest taxonomic level possible) in percent of all sequences in the DNA and RNA datasets (excluding rRNA genes and rRNAs); ordered descending according to DNA counts and supplemented with the remainder of the top eight organisms from the RNA dataset if not already present in the DNA dataset. Please note that no RNA sequences were identified as similar to the SUP05 cluster bacterium at 20 and 40 m with BLASTx-searches against the non-redundant database of NCBI. (PDF) [file pone.0068661.s003.pdf]

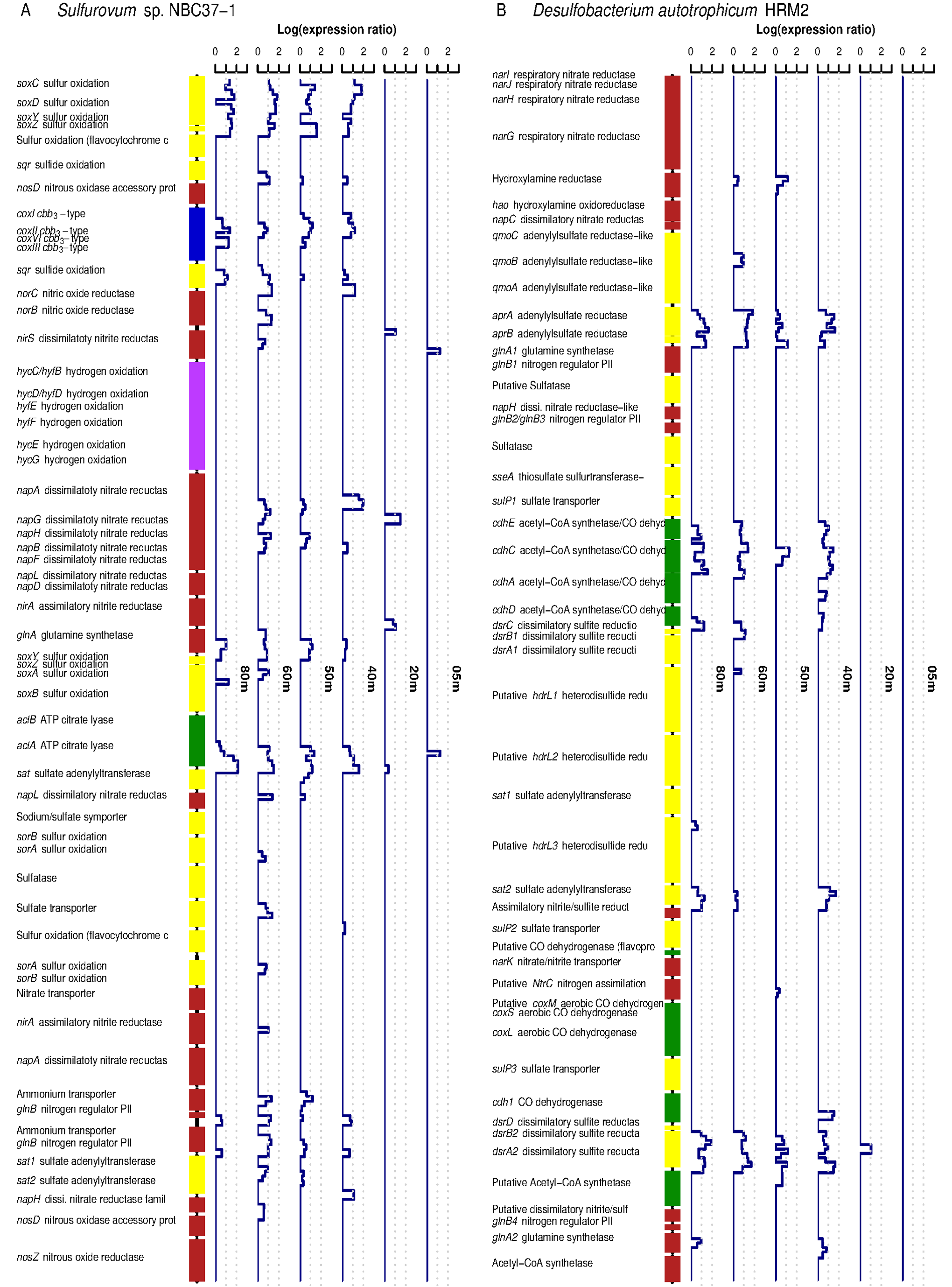

Supplement: Figure S4 — Vertical distribution of sequences recruited onto the genomes of (A) Sulfurovum sp. NBC37- 1 and (B) Desulfobacterium autotrophicum HRM2. Shown are selected genes encoding for enzymes involved in oxygen- (blue), sulfur- (yellow), nitrogen- (red), carbon- (green) and hydrogen-metabolism (purple) in the corresponding order of the genomes. The y-axis depicts the log of the expression-ratio, a measure for the selective enrichment of transcripts over the corresponding gene, normalized to the total pool of protein-coding sequences. A list of the start and end position of each gene and the full name of the corresponding enzyme are shown in Table S3. (TIFF) [file pone.0068661.s004.tif]

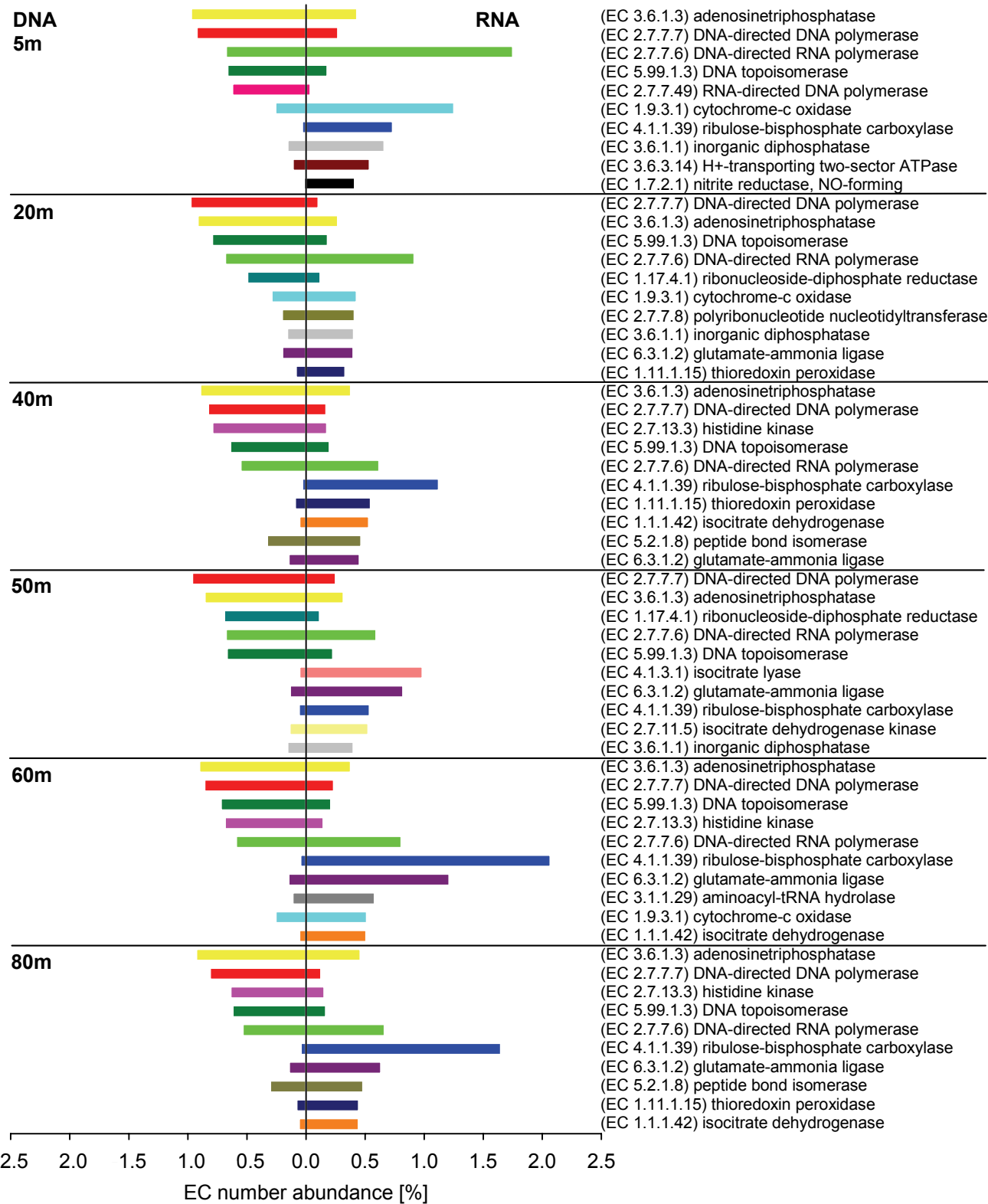

Supplement: Figure S5 — Vertical distribution of the most abundant functional assignments. Shown are the top five most abundant EC numbers in percent of all protein-coding sequences in the DNA and RNA datasets; ordered descending according to the DNA counts and supplemented with the remainder of the top five EC numbers from the RNA dataset if not already present in the DNA dataset. Please note that the data presented here is based only on EC number- and Pfam-assignments; BLAST-hits are not included. (PDF) [file pone.0068661.s005.pdf]
