# Supplementary material for: Giant Hydrogen Sulfide Plume in the Oxygen Minimum Zone off Peru Supports Chemolithoautotrophy
Source: PLoS One. 2013 Aug 21;8(8):e68661. doi: 10.1371/journal.pone.0068661 (PMC3749208; doi:10.1371/journal.pone.0068661)
Supplement: Table S1 — Sequencing statistics. aas obtained with Cd-hit. bas obtained by BLASTn-searches against the SILVA database. cas obtained by BLASTx-searches against the non-redundant database of NCBI and by scans with profile hidden Markov models of the ModEnzA EC groups and of the Pfam protein families. daverage. (DOC) [file pone.0068661.s006.doc]

|  | Number of base pairs | Number of sequences | Average length (bp) | Unique sequences^a^ | Ribosomal sequences^b^ | Protein-coding sequnces^c^ | Not identified sequences |
| --- | --- | --- | --- | --- | --- | --- | --- |
| 5m - DNA | 122,220,795 | 315,414 | 387 | 277,246 | 1,036 | 105,802 | 208,576 |
| 5m - RNA | 127,903,752 | 354,281 | 364 | 99,476 | 320,329 | 5,599 | 28,353 |
| 20m - DNA | 100,015,865 | 265,343 | 380 | 213,668 | 644 | 138,433 | 126,266 |
| 20m - RNA | 71,241,493 | 204,205 | 349 | 83,474 | 155,959 | 17,605 | 30,641 |
| 40m - DNA | 118,370,710 | 296,291 | 402 | 240,667 | 983 | 175,639 | 119,669 |
| 40m - RNA | 100,799,133 | 250,292 | 403 | 99,238 | 188,480 | 35,500 | 26,312 |
| 50m - DNA | 124,119,866 | 312,453 | 397 | 249,195 | 913 | 178,679 | 132,861 |
| 50m - RNA | 101,156,732 | 259,169 | 390 | 106,424 | 153,495 | 65,181 | 40,493 |
| 60m - DNA | 138,696,504 | 347,136 | 399 | 287,253 | 1,189 | 208,405 | 137,542 |
| 60m - RNA | 100,252,938 | 245,161 | 408 | 103,656 | 142,620 | 63,450 | 39,091 |
| 80m - DNA | 154,015,471 | 352,131 | 437 | 313,608 | 1,158 | 218,562 | 132,411 |
| 80m - RNA | 97,749,062 | 247,851 | 394 | 100,443 | 177,068 | 36,809 | 33,974 |
| Total - DNA | 757,439,211 | 1,888,768 | 400 | 263,606^d^ | 5,923 | 1,025,520 | 857,325 |
| Total - RNA | 599,103,110 | 1,560,959 | 385 | 98,785^d^ | 1,137,951 | 224,144 | 198,864 |
