# Supplementary material for: Giant Hydrogen Sulfide Plume in the Oxygen Minimum Zone off Peru Supports Chemolithoautotrophy
Source: PLoS One. 2013 Aug 21;8(8):e68661. doi: 10.1371/journal.pone.0068661 (PMC3749208; doi:10.1371/journal.pone.0068661)
Supplement: Table S2 — Metabolic and taxonomic evenness and diversity in all protein-coding sequences. A collection of all EC number- and Pfam-assignments was used to determine the metabolic diversity, while the taxonomic diversity was calculated using all hits from BLASTx-searches. Shown are the evenness and the diversity (inverse of the Simpson's index) for both the metagenomic and metatranscriptomic datasets. (DOC) [file pone.0068661.s007.doc]

|  | Metabolism | | | | Taxonomy | | | |
| --- | --- | --- | --- | --- | --- | --- | --- | --- |
|  | DNA | | RNA | | DNA | | RNA | |
| Depths | Evenness | Diversity | Evenness | Diversity | Evenness | Diversity | Evenness | Diversity |
| 5m | 0.37 | 130.80 | 0.15 | 36.25 | 0.15 | 5.28 | 0.18 | 5.71 |
| 20m | 0.46 | 163.98 | 0.24 | 66.72 | 0.12 | 4.21 | 0.15 | 5.15 |
| 40m | 0.42 | 157.81 | 0.26 | 66.51 | 0.17 | 6.00 | 0.17 | 5.92 |
| 50m | 0.38 | 132.64 | 0.25 | 64.09 | 0.10 | 3.67 | 0.10 | 3.53 |
| 60m | 0.48 | 172.15 | 0.20 | 46.41 | 0.12 | 4.25 | 0.10 | 3.53 |
| 80m | 0.36 | 128.77 | 0.18 | 42.95 | 0.14 | 4.97 | 0.12 | 4.35 |
