# Supplementary material for: Giant Hydrogen Sulfide Plume in the Oxygen Minimum Zone off Peru Supports Chemolithoautotrophy
Source: PLoS One. 2013 Aug 21;8(8):e68661. doi: 10.1371/journal.pone.0068661 (PMC3749208; doi:10.1371/journal.pone.0068661)
Supplement: Table S3 — Genomic regions of the sequences recruited onto proteobacterial genomes as plotted in Figures 5 and S4. Shown are the start and end position of each gene and the corresponding enzyme name for the uncultured SUP05 cluster bacterium, Candidatus Ruthia magnifica str. Cm, Candidatus Vesicomyosocius okutanii HA, Sulfurovum sp. NBC37- 1 and Desulfobacterium autotrophicum HRM2. (DOC) [file pone.0068661.s008.doc]

| **Uncultured SUP05 cluster bacterium** |
| --- |
| 23505, 25394, *soxB* sulfur oxidation |
| 107746, 109647, *aprA* adenylylsulfate reductase |
| 109864, 110197, *aprB* adenylylsulfate reductase |
| 111266, 112474, *sat* sulfate adenylyltransferase |
| 173044, 173866, *nirK* dissi. nitrite reductase |
| 210707, 211918, Ammonia transport |
| 211929, 212276, *glnB* nitrogen regulator PII |
| 332050, 333357, *fccB* sulfur oxidation |
| 333370, 333957, *fccA* sulfur oxidation |
| 335190, 335672, *napF* dissi. nitrate reductase |
| 335722, 336267, *napB* dissi. nitrate reductase |
| 336492, 338819, *napA* dissi. nitrate reductase |
| 338925, 339794, *napH* dissi. nitrate reductase |
| 339791, 340633, *napG* dissi. nitrate reductase |
| 340643, 340897, *napD* dissi. nitrate reductase |
| 349545, 351011, *narK* nitrate/nitrite transport |
| 351026, 353407, *nirB* assi. nitrite reductase |
| 359192, 359527, *dsrC*-like protein |
| 359743, 360435, *narI* dissi. nitrate reductase |
| 360447, 360962, *narJ* dissi. nitrate reductase |
| 360972, 362522, *narH* dissi. nitrate reductase |
| 362538, 366281, *narG* dissi. nitrate reductase |
| 366479, 368092, *narK* nitrate/nitrite transport |
| 368124, 369533, *narK2* nitrate/nitrite transport |
| 370535, 372781, *cbbO* RuBisCo (ribulose-bisphosphate carboxylase/oxygenase) regulator |
| 372797, 373447, *cbbQ* RuBisCo (ribulose-bisphosphate carboxylase/oxygenase) regulator |
| 373711, 375093, RuBisCo (ribulose-bisphosphate carboxylase/oxygenase) |
| 448830, 449651, *soxA* sulfur oxidation |
| 449675, 449977, *soxZ* sulfur oxidation |
| 450011, 450454, *soxY* sulfur oxidation |
| 450465, 450812, *soxX* sulfur oxidation |
| 493067, 494722, Sulfate permease family protein |
| 514544, 515821, Ammonia permease |
| 515832, 516170, *glnB* nitrogen regulator PII |
| 588232, 589524, *sqr* (sulfide:quinone oxidoreductase) sulfide oxidation |
| 624824, 625366, *dsrA* sulfur oxidation |
| 625368, 626129, *dsrA* sulfur oxidation |
| 626165, 627238, *dsrB* sulfur oxidation |
| 627253, 627654, *dsrE* sulfur oxidation |
| 627656, 628060, *dsrF* sulfur oxidation |
| 628073, 628369, *dsrH*-like protein |
| 628396, 628719, *dsrC* sulfur oxidation |
| 628795, 629577, *dsrM* sulfur oxidation |
| 629579, 630946, *dsrK* sulfur oxidation |
| 774165, 775056, Sulfate permease |
| 868785, 870200, *glnA* glutamine synthetase |
| 1173500, 1174903, *norB* nitric oxide reductase |
| 1174900, 1175574, *norC* nitric oxide reductase |
|  |
| ***Candidatus* Ruthia magnifica str. Cm** |
| 39283, 40350, *coxII* (cytochrome c oxidase) |
| 40373, 41989, *coxI* (cytochrome c oxidase) |
| 42080, 42613, *cox* (cytochrome c oxidase) assembly protein |
| 42631, 43515, *coxIII* (cytochrome c oxidase) |
| 44618, 46036, *coxI* (cytochrome c oxidase), *cbb*_3_-type |
| 46050, 46781, *coxII* (cytochrome c oxidase), *cbb*_3_-type |
| 46945, 47847, *coxIII* (cytochrome c oxidase), *cbb*_3_-type |
| 101492, 102700, *sat* sulfate adenylyltransferase |
| 103798, 104277, *aprB* adenylylsulfate reductase |
| 104277, 106160, *aprA* adenylylsulfate reductase |
| 182253, 184142, *soxB* sulfur oxidation |
| 230535, 230885, *glnB* nitrogen regulator PII |
| 301598, 301936, *glnB* nitrogen regulator PII |
| 301983, 303221, Ammonium transport |
| 307708, 308055, *glnB* nitrogen regulator PII |
| 344806, 345219, Thiosulfate sulfurtransferase |
| 543750, 545165, *glnA* glutamine synthetase |
| 676385, 676711, *dsrC* family protein |
| 739720, 741186, Nitrate/nitrite transport |
| 741202, 743577, *nirB* assi. nitrite reductase |
| 752989, 753798, *cbbQ* RuBisCo (ribulose-bisphosphate carboxylase/oxygenase) regulator |
| 753897, 755279, RuBisCo (ribulose-bisphosphate carboxylase/oxygenase) |
| 858411, 859226, *soxA* sulfur oxidation protein |
| 859254, 859556, *soxZ* sulfur oxidation protein |
| 859589, 860032, *soxY* sulfur oxidation protein |
| 860043, 860390, *soxX* sulfur oxidation protein |
| 916759, 917961, *nrfD* polysulfide reductase |
| 922717, 923490, *dsrM*-like protein* |
| 923564, 923887, *dsrC* family protein |
| 923914, 924210, *dsrH* family protein |
| 924223, 924621, *dsrE* family protein |
| 924623, 925024, *dsrE* family protein |
| 925037, 926110, *dsrB* sulfur oxidation |
| 926183, 927484, *dsrA* sulfur oxidation |
| 939512, 939802, *dsrC*-related protein |
| 1130921, 1132216, *sqr* (sulfide:quinone oxidoreductase) sulfide oxidation |
|  |
| ***Candidatus* Vesicomyosocius okutanii HA** |
| 34428, 35537, *coxII* (cytochrome c oxidase) |
| 35563, 37164, *coxI* (cytochrome c oxidase) |
| 37255, 37788, *cox* (cytochrome c oxidase) assembly protein |
| 37806, 38690, *coxIII* (cytochrome c oxidase) |
| 39798, 41216, *coxI* (cytochrome c oxidase), *cbb*_3_-type |
| 41231, 41962, *coxII* (cytochrome c oxidase), *cbb*_3_-type |
| 41962, 42102, *coxIV* (cytochrome c oxidase), *cbb*_3_-type |
| 42121, 43023, *coxIII* (cytochrome c oxidase), *cbb*_3_-type |
| 98093, 99301, *sat* sulfate adenylyltransferase |
| 100417, 100896, *aprB* adenylylsulfate reductase |
| 100896, 102779, *aprA* adenylylsulfate reductase |
| 172596, 174485, *soxB* sulfur oxidation |
| 211500, 211850, *glnB* nitrogen regulator PII |
| 264163, 264501, *glnB* nitrogen regulator PII |
| 264491, 265786, Ammonium transport |
| 270118, 270465, *glnB* nitrogen regulator PII |
| 473999, 475414, *glnA* glutamine synthetase |
| 600392, 600733, *dsrC*-like protein |
| 660239, 661705, *narK* nitrate transport |
| 661708, 664077, *nirB* assi. nitrite reductase |
| 669765, 670445, *narI* resp. nitrate reductase |
| 670461, 671117, *narJ* resp. nitrate reductase |
| 671114, 672655, *narH* resp. nitrate reductase |
| 672655, 676386, *narG* resp. nitrate reductase |
| 678095, 680341, *cbbO* RuBisCo (ribulose-bisphosphate carboxylase/oxygenase) regulator |
| 680357, 681166, *cbbQ* RuBisCo (ribulose-bisphosphate carboxylase/oxygenase) regulator |
| 681254, 682636, RuBisCo (ribulose-bisphosphate carboxylase/oxygenase) |
| 770792, 771607, *soxA* sulfur oxidation |
| 771635, 771937, *soxZ* sulfur oxidation |
| 771971, 772414, *soxY* sulfur oxidation |
| 772425, 772772, *soxX* sulfur oxidation |
| 790563, 791690, *sqr* (sulfide:quinone oxidoreductase) sulfide oxidaton |
| 817196, 817537, *dsrR* sulfur oxidation |
| 817534, 818910, *dsrN* sulfur oxidation |
| 818938, 820140, *dsrP* sulfur oxidation |
| 820166, 820897, *dsrO* sulfur oxidation |
| 820894, 821277, *dsrJ* sulfur oxidation |
| 821307, 823271, *dsrL* sulfur oxidation |
| 823327, 824892, *dsrK* sulfur oxidation |
| 824894, 825667, *dsrM* sulfur oxidation |
| 825744, 826067, *dsrC* sulfur oxidation |
| 826094, 826390, *dsrH* sulfur oxidation |
| 826402, 826806, *dsrF* sulfur oxidation |
| 826810, 827211, *dsrE* sulfur oxidation |
| 827224, 828297, *dsrB* sulfur oxidation |
| 828373, 829674, *dsrA* sulfur oxidation |
| 839956, 840246, *dsrC* sulfur oxidation |
| 995954, 997240, *sqr* (sulfide:quinone oxidoreductase) sulfide oxidation |
|  |
| ***Sulfurovum* sp. NBC37- 1** |
| 53157, 54473, *soxC* sulfur oxidation |
| 54454, 55626, *soxD* sulfur oxidation |
| 55666, 56178, *soxY* sulfur oxidation |
| 56258, 56560, *soxZ* sulfur oxidation |
| 60805, 62172, Sulfur oxidation (flavocytochrome c) |
| 73399, 74571, *sqr* (sulfide:quinone oxidoreductase) sulfide oxidation |
| 159880, 161109, *nosD* nitrous oxidase accessory protein |
| 181741, 183207, *coxI* (cytochrome c oxidase), *cbb*_3_-type |
| 183219, 183908, *coxII* (cytochrome c oxidase), *cbb*_3_-type |
| 183914, 184132, *coxVI* (cytochrome c oxidase), *cbb*_3_-type |
| 184129, 185019, *coxIII* (cytochrome c oxidase), *cbb*_3_-type |
| 194939, 196390, *sqr* (sulfide:quinone oxidoreductase) sulfide oxidation |
| 242443, 243114, *norC* nitric oxide reductase |
| 243104, 244549, *norB* nitric oxide reductase |
| 244879, 246594, *nirS* dissimilatoty nitrite reductase |
| 259164, 261047, *hycC*/*hyfB* hydrogen oxidation |
| 261044, 261964, *hycD*/*hyfD* hydrogen oxidation |
| 261968, 262561, *hyfE* hydrogen oxidation |
| 262558, 263973, *hyfF* hydrogen oxidation |
| 263970, 265337, *hycE* hydrogen oxidation |
| 265334, 265840, *hycG* hydrogen oxidation |
| 300766, 303603, *napA* dissimilatoty nitrate reductase |
| 303608, 304441, *napG* dissimilatoty nitrate reductase |
| 304441, 305244, *napH* dissimilatoty nitrate reductase |
| 305263, 306231, *napB* dissimilatoty nitrate reductase |
| 306231, 306743, *napF* dissimilatoty nitrate reductase |
| 307192, 308145, *napL* dissimilatoty nitrate reductase |
| 308156, 308515, *napD* dissimilatoty nitrate reductase |
| 313115, 314764, *nirA* assimilatory nitrite reductase |
| 366880, 368310, *glnA* glutamine synthetase |
| 503764, 504243, *soxY* sulfur oxidation |
| 504299, 504637, *soxZ* sulfur oxidation |
| 504641, 505402, *soxA* sulfur oxidation |
| 505412, 507187, *soxB* sulfur oxidation |
| 551581, 552891, *aclB* ATP citrate lyase |
| 552907, 554727, *aclA* ATP citrate lyase |
| 526620, 527798, *sat* sulfate adenylyltransferase |
| 696198, 697148, *napL* dissimilatoty nitrate reductase |
| 993735, 995081, Sodium/sulfate symporter |
| 1126255, 1126572, *sorB* sulfur oxidation |
| 1126584, 1127789, *sorA* sulfur oxidation |
| 1184827, 1186743, Sulfatase |
| 1201373, 1202956, Sulfate transporter |
| 1378920, 1380239, Sulfur oxidation (flavocytochrome c) |
| 1521996, 1523204, *sorA* sulfur oxidation |
| 1523201, 1523530, *sorB* sulfur oxidation |
| 1544689, 1545969, Nitrate transporter |
| 1546196, 1548115, *nirA* assimilatory nitrite reductase |
| 1554543, 1556867, *napA* dissimilatoty nitrate reductase |
| 1695756, 1697063, Ammonium transporter |
| 1697198, 1697539, *glnB* nitrogen regulator PII |
| 1698115, 1699287, Ammonium transporter |
| 1699300, 1699641, *glnB* nitrogen regulator PII |
| 1774429, 1775868, *sat1* sulfate adenylyltransferase |
| 1775870, 1776781, *sat2* sulfate adenylyltransferase |
| 2265182, 2266093, *napH* dissi. nitrate reductase family protein |
| 2268064, 2269287, *nosD* nitrous oxidase accessory protein |
| 2270271, 2272871, *nosZ* nitrous oxide reductase |
|  |
| ***Desulfobacterium autotrophicum* HRM2** |
| 79712, 80398, *narI* respiratory nitrate reductase |
| 80428, 80994, *narJ* respiratory nitrate reductase |
| 81017, 82438, *narH* respiratory nitrate reductase |
| 82428, 86126, *narG* respiratory nitrate reductase |
| 107440, 109101, Hydroxylamine reductase |
| 111904, 113301, *hao* hydroxylamine oxidoreductase |
| 113343, 113867, *napC* dissimilatory nitrate reductase |
| 513380, 514555, *qmoC* adenylylsulfate reductase-like protein |
| 514591, 516927, *qmoB* adenylylsulfate reductase-like protein |
| 516935, 518209, *qmoA* adenylylsulfate reductase-like protein |
| 518490, 520451, *aprA* adenylylsulfate reductase |
| 520508, 520945, *aprB* adenylylsulfate reductase |
| 765848, 767260, *glnA1* glutamine synthetase |
| 767296, 767634, *glnB1* nitrogen regulator PII |
| 1089653, 1091512, Putative sulfatase |
| 1092844, 1093680, *napH* dissi. nitrate reductase-like protein |
| 1107061, 1107788, *glnB2*/*glnB3* nitrogen regulator PII |
| 1190379, 1192217, Sulfatase |
| 1450969, 1452849, *sseA* thiosulfate sulfurtransferase-like protein |
| 1515688, 1516887, *sulP1* sulfate transporter |
| 1907545, 1908885, *cdhE* acetyl-CoA synthetase/CO dehydrogenase |
| 1908976, 1911189, *cdhC* acetyl-CoA synthetase/CO dehydrogenase |
| 1911259, 1913295, *cdhA* acetyl-CoA synthetase/CO dehydrogenase |
| 1913836, 1915137, *cdhD* acetyl-CoA synthetase/CO dehydrogenase |
| 2487823, 2488140, *dsrC* dissimilatory sulfite reduction |
| 2488276, 2489121, *dsrB1* dissimilatory sulfite reduction |
| 2489140, 2490195, *dsrA1* dissimilatory sulfite reduction |
| 2491674, 2496122, Putative *hdrL1* heterodisulfide reductase |
| 2577948, 2581370, Putative *hdrL2* heterodisulfide reductase |
| 2872705, 2874399, *sat1* sulfate adenylyltransferase |
| 3505532, 3509995, Putative *hdrL3* heterodisulfide reductase |
| 3538111, 3539388, *sat2* sulfate adenylyltransferase |
| 3795726, 3796382, Assimilatory nitrite/sulfite reductase |
| 3797720, 3799492, *sulP2* sulfate transporter |
| 3847454, 3847750, Putative CO dehydrogenase (flavoprotein) |
| 3913360, 3914553, *narK* nitrate/nitrite transporter |
| 4187991, 4189358, Putative *ntrC* nitrogen assimilation regulator |
| 4266961, 4267788, Putative *coxM* aerobic CO dehydrogenase |
| 4267785, 4268255, *coxS* aerobic CO dehydrogenase |
| 4268246, 4270555, *coxL* aerobic CO dehydrogenase |
| 4540182, 4542308, *sulP3* sulfate transporter |
| 4606186, 4608150, *cdh1* CO dehydrogenase |
| 4749506, 4749763, *dsrD* dissimilatory sulfite reductase |
| 4749859, 4751007, *dsrB2* dissimilatory sulfite reductase |
| 4751024, 4752349, *dsrA2* dissimilatory sulfite reductase |
| 4872760, 4874640, *cdh2* CO dehydrogenase |
| 5045877, 5048279, Putative Acetyl-CoA synthetase |
| 5061740, 5062543, Putative dissimilatory nitrite/sulfite reductase |
| 5130721, 5131059, *glnB4* nitrogen regulator PII |
| 5142825, 5144153, *glnA2* glutamine synthetase |
| 5322777, 5324543, Acetyl-CoA synthetase |
